# Genomic prediction using information across years with epistatic models and dimension reduction via haplotype blocks

**DOI:** 10.1371/journal.pone.0282288

**Published:** 2023-03-31

**Authors:** Elaheh Vojgani, Armin C. Hölker, Manfred Mayer, Chris-Carolin Schön, Henner Simianer, Torsten Pook

**Affiliations:** 1 Center for Integrated Breeding Research, Animal Breeding and Genetics Group, University of Goettingen, Goettingen, Germany; 2 Plant Breeding, TUM School of Life Sciences Weihenstephan, Technical University of Munich, Freising, Germany; KGUT: Graduate University of Advanced Technology, ISLAMIC REPUBLIC OF IRAN

## Abstract

The importance of accurate genomic prediction of phenotypes in plant breeding is undeniable, as higher prediction accuracy can increase selection responses. In this regard, epistasis models have shown to be capable of increasing the prediction accuracy while their high computational load is challenging. In this study, we investigated the predictive ability obtained in additive and epistasis models when utilizing haplotype blocks versus pruned sets of SNPs by including phenotypic information from the last growing season. This was done by considering a single biological trait in two growing seasons (2017 and 2018) as separate traits in a multi-trait model. Thus, bivariate variants of the Genomic Best Linear Unbiased Prediction (GBLUP) as an additive model, Epistatic Random Regression BLUP (ERRBLUP) and selective Epistatic Random Regression BLUP (sERRBLUP) as epistasis models were compared with respect to their prediction accuracies for the second year. The prediction accuracies of bivariate GBLUP, ERRBLUP and sERRBLUP were assessed with eight phenotypic traits for 471/402 doubled haploid lines in the European maize landrace Kemater Landmais Gelb/Petkuser Ferdinand Rot. The results indicate that the obtained prediction accuracies are similar when utilizing a pruned set of SNPs or haplotype blocks, while utilizing haplotype blocks reduces the computational load significantly compared to the pruned sets of SNPs. The number of interactions considered in the model was reduced from 323.5/456.4 million for the pruned SNP panel to 4.4/5.5 million in the haplotype block dataset for Kemater and Petkuser landraces, respectively. Since the computational load scales linearly with the number of parameters in the model, this leads to a reduction in computational time of 98.9% from 13.5 hours for the pruned set of markers to 9 minutes for the haplotype block dataset. We further investigated the impact of genomic correlation, phenotypic correlation and trait heritability as factors affecting the bivariate models’ prediction accuracy, identifying the genomic correlation between years as the most influential one. As computational load is substantially reduced, while the accuracy of genomic prediction is unchanged, the here proposed framework to use haplotype blocks in sERRBLUP provided a solution for the practical implementation of sERRBLUP in real breeding programs. Furthermore, our results indicate that sERRBLUP is not only suitable for prediction across different locations, but also for the prediction across growing seasons.

## Introduction

In plant breeding, genomic prediction has become a regular tool [1,2] which enables the optimization of phenotyping costs of breeding programs [3]. The importance of genomic prediction of phenotypes is not restricted to plants. Livestock [4] and human research [5] also are widely developed in this regard. In the context of plant and animal breeding, accurately predicting phenotypic traits is of special importance, since raising all animals and growing all crops to measure their performances requires a considerable amount of money under limited resources [6].

Several statistical models have been compared over the last decades in the context of prediction accuracy. Among the additive models, genomic best linear unbiased prediction (GBLUP) [7,8] has been widely used due to its high robustness, computing speed and superiority in predictive ability to alternative prediction models like Bayesian methods, especially in small reference populations [9–12]. Furthermore, the inclusion of genotype × environment interaction into additive genomic prediction models can result in an increase in prediction accuracy [13,14]. Such approaches allow borrowing information across environments which potentially leads to higher accuracy in phenotype prediction in multi environment models [15]. In fact, multivariate mixed models were originally proposed in the context of animal breeding [16] with the purpose of modeling the genomic correlation among traits, longitudinal data, and modeling genotype by environment interactions across multiple years or environments [11,17,18]. A multivariate GBLUP model was reported to have higher prediction accuracy than univariate GBLUP when the genetic correlations were medium (0.6) or high (0.9) [11]. It was also shown that aggregating the phenotypic data over years to train the model and predict the performance of lines in the following years is a possible approach that can improve prediction accuracy [19,20].

In addition, the inclusion of epistasis, defined as the interaction between loci [21,22], into the genomic prediction model results in more accurate phenotype prediction [6,23–26] due to the considerable contribution of epistasis in genetic variation of quantitative traits [25]. In this context, several statistical models have been proposed. Extended genomic best linear unbiased prediction (EG-BLUP, [27]) and categorical epistasis (CE, [28]) models are using a marker-based epistatic relationship matrix that can be constructed in a highly efficient manner. It was shown that the CE model is as good as or better than EG-BLUP without having undesirable features of EG-BLUP such as coding-dependency [28].

Further, it was shown that the accuracy of the epistasis genomic prediction model can be increased in one environment by variable selection in another environment [6]. In this approach, the full epistasis model was reduced to a model with a subset of the largest epistatic interaction effects, resulting in an increase in predictive ability [6], through borrowing information across environments. Vojgani et al. (2021) [26] showed that the prediction accuracy can be increased even further by selecting the interactions with the highest absolute effect sizes or variances in the epistasis model. The resulting higher computational needs were offset by the development of a highly efficient software package “EpiGP” [29], performing computations in a bit-wise manner [30]. This enabled predictions with data sets of practically relevant size across environments, both with respect to sample size and number of markers [26]. Since the number of interactions to be considered increases quadratically with the number of variables included, the computational load of methods such as EpiGP can quickly get out of control, since a model with 600,000 SNPs, as occurs in high-density arrays [31,32], would result in more than a hundred billion interactions to be considered. The most common variable reduction method used here is LD pruning [33], but new linkage-based haplotyping methods [34] to further reduce the dimensionality of genomic data without much loss of information [35] are worth exploring.

The aim of this study is to compare the obtained prediction accuracies of bivariate genomic prediction models incorporating epistatic interactions through borrowing information across years when utilizing haplotype blocks versus pruned sets of SNPs, as a novel approach in epistasis models. As the accuracy of marker-based genomic prediction of phenotypes was shown to be increased by both borrowing information across environments and/or years [11,20] and the inclusion of epistasis into the prediction model [6,36], we combine these two approaches in both SNP-based and haplotype-based genomic prediction models to make the best use of the available information. We further find the optimum proportion of epistatic interactions of pruned sets of SNPs and haplotype blocks to be kept in the model in the variable selection step across years and compare their obtained predictive abilities. We further study structural factors in the data which might affect the predictive ability across years.

## Materials and methods

### Data used for analysis

A set of 948 doubled haploid lines of the European maize landraces Kemater Landmais Gelb (KE, Austria, 516 lines) and Petkuser Ferdinand Rot (PE, Germany, 432 lines) were genotyped with the 600 k Affymetrix® Axiom® Maize Array [32], which were utilized separately for genomic prediction of phenotypes.

After quality filtering and imputation, 910 DH lines remained (501 lines in KE and 409 lines in PE) and the panel of markers was reduced to 501,124 markers [37]. Additionally, loci that were in high level of pairwise linkage disequilibrium (LD) were removed [38] through linkage disequilibrium based SNP pruning with PLINK v1.07 [33,39] with the parameters 50, 5 and 2 indicating the SNPs window size, the number of SNPs by which the SNP window is shifted and the variance inflation factor, respectively. This resulted in a data panel containing 25’437 SNPs for KE and 30’212 SNPs for PE [36]. Note that even a panel of 25’000 SNPs results in more than 1 billion SNP interactions to account for. Therefore, in order to have a further variable reduction, haplotype blocks as a combination of closely linked markers, which has shown to be an alternative approach for genomic prediction improving the prediction accuracy [40,41], were generated from the full panel of markers with the software HaploBlocker [34] using default settings. This resulted in a data panel containing 2’972 haplotype blocks in KE and 3’330 haplotype blocks in PE. Monte Carlo analysis of variance [35] suggests that 99.2% / 98.2% of the variance of the pruned SNP dataset is explained by haplotype blocks in KE / PE.

Out of 910 genotyped lines, only 873 DH lines were phenotyped (471 lines in KE and 402 lines in PE). Einbeck (EIN, Germany), Roggenstein (ROG, Germany), Golada (GOL, Spain) and Tomeza (TOM, Spain) were the four locations where these lines were phenotyped for a series of traits in both 2017 and 2018.

The means, standard deviations, maximum and minimum values of studied phenotypic traits in 2017 and 2018 in each landrace are compared in Table 1 which were derived from the Best Linear Unbiased Estimations (BLUEs) of the genotype mean for each phenotypic trait by Hölker *et al*. (2019). The comparison of the respective detailed values for each trait in each environment and landrace in 2017 and 2018 are illustrated in the (S1 Table). Vi in phenotypic traits represents the vegetative growth stage when *i* leaf collars are visible based on the leaf collar method of the corn growth [42]. Early vigour at V3 stage (EV_V3), female flowering (FF) and root lodging (RL) were not phenotyped in all four environments for both years. EV_V3 was not phenotyped in EIN in 2018, FF was not phenotyped in GOL in 2017 and RL was not phenotyped in TOM and GOL in both 2017 and 2018.

**Table 1:** Phenotypic trait description and the mean, minimum, maximum and standard deviation of the BLUEs for each phenotypic trait in KE and PE landraces in the years 2017 and 2018.

Trait	Definition	Landrace	Year	Mean	Minimum	Maximum	Standard deviation
EV_V3	Early vigour at V3 stage scored on scale from 1 (very poor early vigour) to 9 (very high early vigour)	KE	2017 2018	4.94 5.06	0.78 0.32	9.00 8.67	1.35 1.33
		PE	2017 2018	5.57 5.47	1.00 1.38	9.03 8.93	1.20 1.13
EV _V4	Early vigour at V4 stage scored on scale from 1 (very poor early vigour) to 9 (very high early vigour)	KE	2017 2018	4.84 5.08	0.67 0.96	8.29 8.65	1.30 1.30
		PE	2017 2018	5.45 5.25	0.93 1.63	8.49 9.07	1.15 1.19
EV _V6	Early vigour at V6 stage scored on scale from 1 (very poor early vigour) to 9 (very high early vigour)	KE	2017 2018	5.13 5.54	0.54 1.07	8.75 9.60	1.31 1.35
		PE	2017 2018	5.64 5.38	0.84 1.07	8.39 9.68	1.12 1.29
PH_V4	Mean plant height of three plants of the plot at V4 stage in cm	KE	2017 2018	33.10 42.01	6.90 8.48	88.24 89.24	13.95 16.47
		PE	2017 2018	38.01 46.19	11.89 16.14	95.30 93.20	14.96 17.78
PH_V6	Mean plant height of three plants of the plot at V6 stage in cm	KE	2017 2018	62.03 92.27	8.34 21.90	127.54 173.66	19.95 21.04
		PE	2017 2018	69.84 97.80	14.78 50.37	130.51 169.71	19.26 19.44
PH_final	Final plant height after flowering in cm	KE	2017 2018	139.10 146.04	49.27 35.41	245.00 265.02	27.14 35.74
		PE	2017 2018	124.09 128.08	30.21 35.76	211.14 248.43	24.54 35.99
FF	Days after sowing until female flowering (days until 50% of the plot showed silks)	KE	2017 2018	79.72 76.99	62.45 62.22	102.02 100.14	6.27 6.09
		PE	2017 2018	78.85 76.70	59.10 60.14	101.50 93.96	6.33 6.52
RL	Root lodging score from 1 to 9 (1 = no lodging and 9 = severe lodging)	KE	2017 2018	3.38 1.42	0.59 0.73	9.58 8.52	2.50 0.90
		PE	2017 2018	2.14 1.21	0.03 0.32	9.22 4.69	1.74 0.51

The number of phenotyped lines per year and environment for trait PH_V4, as the main trait in this study, are summarized in Table 2. For EIN and ROG, a higher number of phenotyped lines were generated in 2017. On the contrary, more lines were phenotypes in GOL and TOM in 2018.

**Table 2:** Number of KE and PE lines phenotyped in each location for the years 2017 (blue numbers) and 2018 (red numbers) for trait PH_V4.

	EIN (2017\2018)	ROG (2017\2018)	GOL (2017\2018)	TOM (2017\2018)
**Phenotyped lines in KE**	462\365	461\365	211\222	211\222
**Phenotyped lines in PE**	393\365	390\365	204\240	204\240

### Statistical models for phenotype prediction

We used the bivariate statistical framework as the basis of the genomic prediction models which has been proposed in the recent work by [36]. In this regard, GBLUP, ERRBLUP and sERRBLUP as three different methods described in Vojgani et al. (2021) [36] were used for genomic prediction of phenotypes which differ in dispersion matrices representing their covariance structure of the genetic effects. GBLUP as an additive model is based on a genomic relationship matrix calculated according to VanRaden (2008) [43]. ERRBLUP (Epistatic Random Regression BLUP) as a full epistasis model is based on all pairwise SNP interactions which is generated from a marker combination matrix of dimension number of lines times number of marker combinations, in which the absence (0) or presence (1) of each marker combination for each line is coded. sERRBLUP (selective Epistatic Random Regression BLUP) as a selective epistasis model is based on a selected subset of SNP interactions [26]. Vojgani et al. (2021) [36] proposed estimated effect variances in the training set as the selection criterion of pairwise SNP interactions due to its robustness in predictive ability specifically when only a small proportion of interactions are maintained in the model.

### Assessment of genomic prediction models

GBLUP, ERRBLUP and sERRBLUP models have been assessed via 5-fold cross validation by randomly partitioning the original sample into 5 equal size subsamples in which one subsample was considered as the test set to validate the model, and the remaining 4 subsamples were considered as a joint training set [44]. The 5-fold cross validation scheme was repeated 5 times, and the Pearson correlation between the predicted values and the observed phenotypes in the test set was considered as the predictive ability in each fold of each replicate, which then was averaged across the 25 replicates. In this study, predictive ability was separately assessed for KE and PE for a series of phenotypic traits in four different environments. Besides, we calculated the traits’ prediction accuracies by dividing their predictive abilities by the square root of the respective traits’ heritabilities [45] derived from all environments in both 2017 and 2018 jointly (S2 Table).

Univariate GBLUP within 2018 was assessed by training the model in the same year (2018) as the test set was sampled from. However, bivariate GBLUP, ERRBLUP and sERRBLUP were assessed by training the model with both the training set of the target environment in 2018 and the full dataset of the respective environment in 2017. The interaction selection step in bivariate sERRBLUP is done by first using the complete dataset of the target environment in 2017 to estimate all pairwise SNP interaction effect variances. Then, an epistatic relationship matrix for all lines is constructed based on the subset of top ranked interaction effect variances, which is finally used to predict phenotypes of the target environment test set in 2018 [36].

### Variance component estimation

Variance component estimation in univariate GBLUP was done by EMMREML [46] based on the training set in each run of 5-fold cross validation with 5 replicates. In bivariate models this was done by ASReml-R [47] with the approach specified by Vojgani et al. (2021) [36] for pre estimating the variance components from the full dataset to derive the initial values for the variance components in ASReml models in 100 iterations for each combination. If the variance estimation based on the full set did not converge after 100 iterations, then the estimated variance components at the 100^th^ iteration were extracted as initial values of the bivariate model in the cross-validation step. Afterwards, the model used these values to re-estimate the variance components based on the training set in each run of 5-fold cross validation in 50 iterations. The estimated variance components in the converged models based on the full set deviated only slightly from the estimated variance components based on the training set (S1 Fig). However, the variance component estimations did not converge in all folds of 5-fold cross validation with 5 replicates. In such cases, the initial values were set as the fixed values for the model to predict the breeding values. This approach appears justifiable in the case of non-convergence of the bivariate model, since we show in S2 Fig that the difference between the mean predictive ability of all folds and only the converged folds does not appear to be critical. This difference can get higher as the number of non-converged folds increases. The percentage of converged folds in all studied material is shown in the (S3 Table).

### Genomic correlation estimation

Genomic correlations were estimated from the genetic variances and covariance derived from the ASReml bivariate model based on the full dataset of each location in both 2017 and 2018.

## Results

Comparison of univariate GBLUP, bivariate GBLUP, bivariate ERRBLUP and bivariate sERRBLUP based on pruned set of SNPs and haplotype blocks in PH_V4.

**Fig 1:**
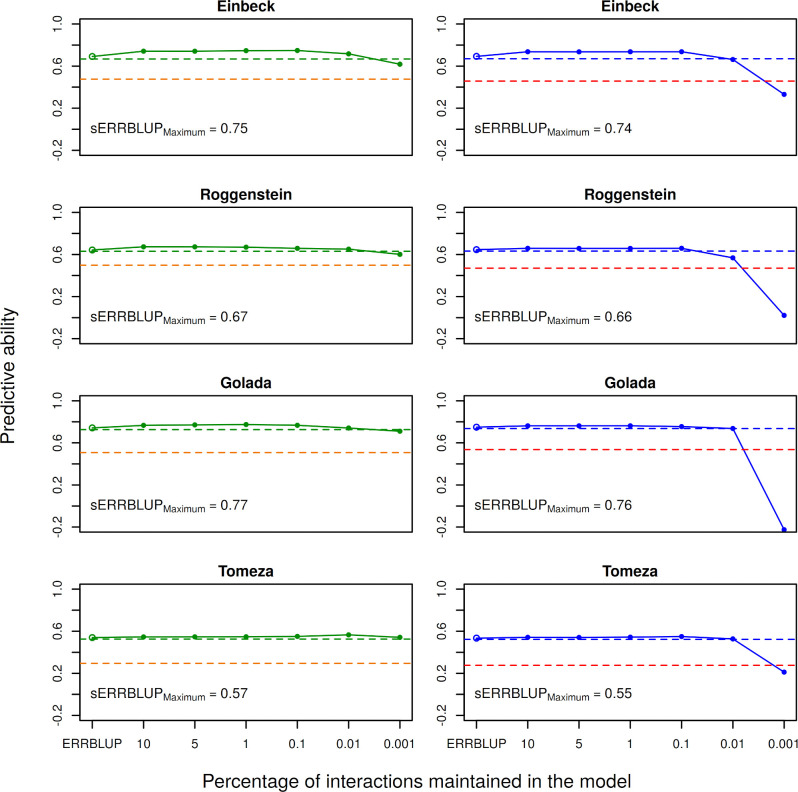
Predictive ability for univariate GBLUP within 2018 (orange and red dashed horizontal line), bivariate GBLUP (green and blue dashed horizontal line), bivariate ERRBLUP (open circle) and bivariate sERRBLUP (filled circles and solid line) for trait PH-V4 in Kemater based on the pruned set of SNPs (left) and haplotype blocks (right). In each plot, the sERRBLUP maximum indicates the maximum predictive ability obtained from bivariate sERRBLUP.

**Fig 2:**
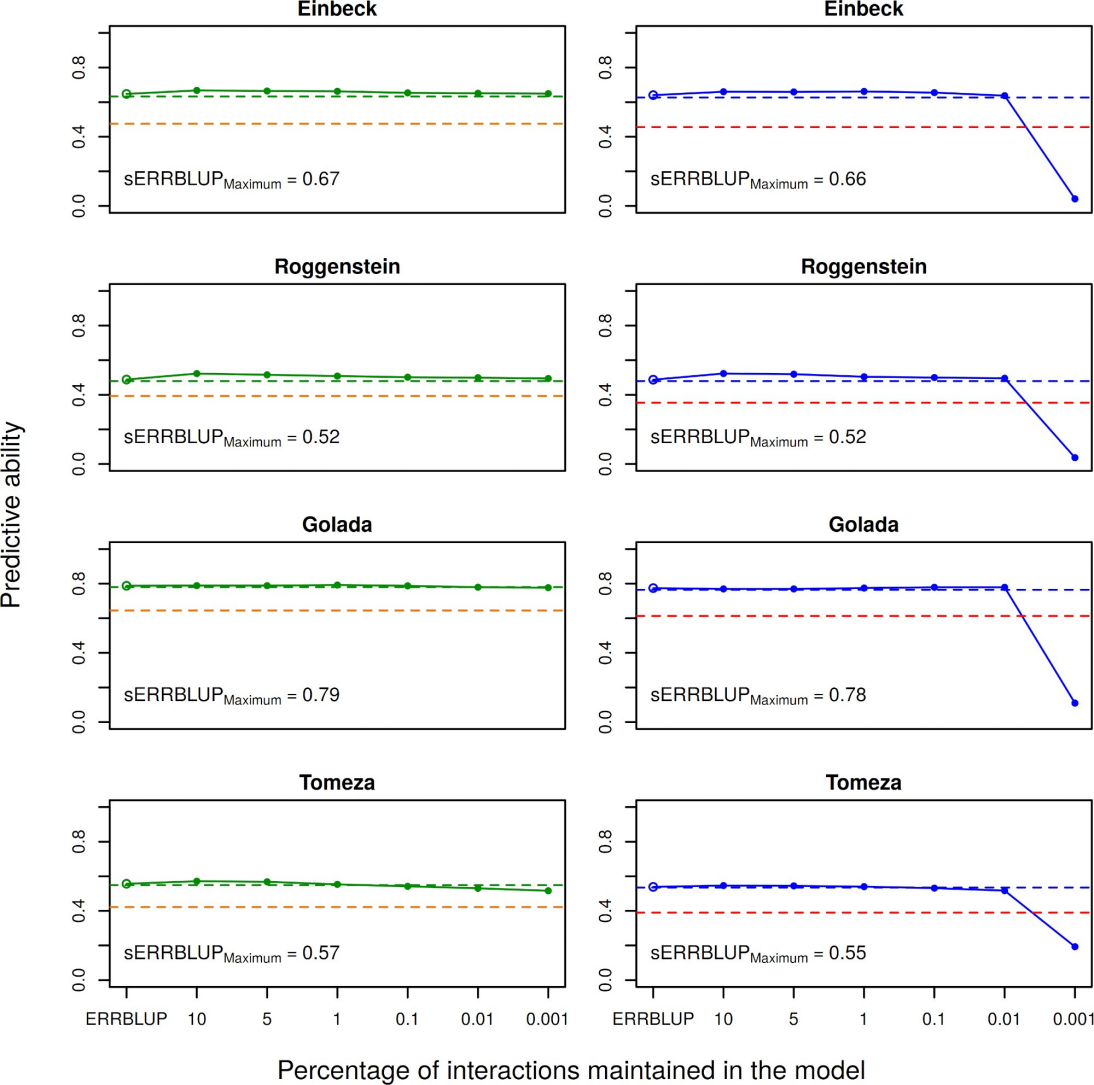
Predictive ability for univariate GBLUP within 2018 (orange and red dashed horizontal line), bivariate GBLUP (green and blue dashed horizontal line), bivariate ERRBLUP (open circle) and bivariate sERRBLUP (filled circles and solid line) for trait PH-V4 in Petkuser based on the pruned sets of SNPs (left) and haplotype blocks (right). In each plot, the sERRBLUP maximum indicates the maximum predictive ability obtained from bivariate sERRBLUP.

In order to compare the haplotype block based predictive abilities with the pruned SNP based predictive abilities in both additive and epistasis models, Figs 1 and 2 are illustrated. First of all, our results confirm the findings of Vojgani et al. (2021) [36] that bivariate models outperform the univariate models as illustrated by the comparison in predictive ability of bivariate GBLUP and univariate GBLUP for the trait PH-V4 in both landraces indicating the superiority of bivariate GBLUP to univariate GBLUP in most cases (see Figs 1 and 2). Among the bivariate genomic prediction models, the predictive ability obtained from bivariate ERRBLUP is almost identical to bivariate GBLUP. As it is expected, this predictive ability increases in bivariate sERRBLUP and the highest gain in accuracy is generally obtained when the top 10 or 5 percent of pairwise SNP interactions are kept in the model, while a too strict selection like using only the top 0.001 percent interactions, results in a decrease in predictive ability (see Figs 1 and 2). The number of interactions maintained in the model for each proportion of interactions are tabulated in the (S4 Table) with the total number of interactions for the full dataset being 125.6 billion interactions compared to 323.5 million and 456.4 million interactions for the pruned dataset and 4.4 million and 5.5 million interactions when using haplotype blocks, respectively for KE and PE. The robustness of the predictive ability depending on the share of selected markers was higher in PE than KE. Moreover, Fig 1 and Fig 2 importantly illustrate the comparison between the predictive abilities obtained from the respective genomic prediction models in KE and PE when utilizing pruned set of SNPs and haplotype blocks. It is shown that the GBLUP, ERRBLUP and sERRBLUP (for the optimum proportions of interactions) predictive abilities are almost identical in both pruned set of SNPs and haplotype blocks. Results when using haplotype blocks were only worse when an extremely low share of interactions (0.01 & 0.001%) was included in the model (Fig 1). As the number of included interactions is then reduced to less than 1’000 (S4 Table) these should not reflect practically relevant parameter settings. Similar patterns are observed across a series of other traits for bivariate models which are shown in the (S3–S16 Figs). Additionally, the predictive ability of univariate GBLUP by training the model on the average phenotypic values of both 2017 and 2018, when utilizing pruned set of SNPs, was evaluated for a series of phenotypic traits, which yielded quite similar predictive ability as obtained with univariate GBLUP within year 2018 or worse in some cases (S5 Table (KE) and S6 Table (PE)).

### Correlation between prediction accuracy and the genomic correlation in bivariate models

**Fig 3:**
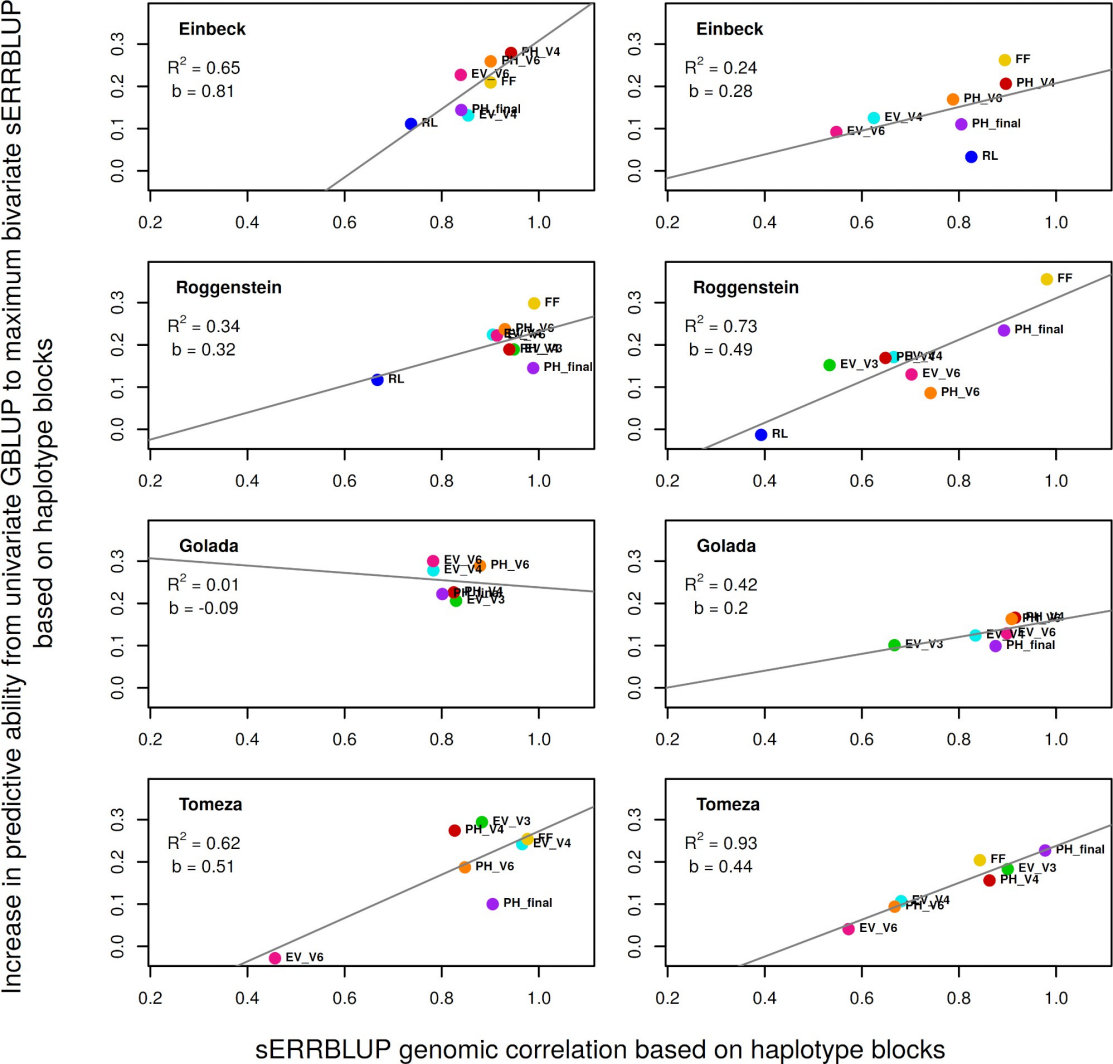
Regression of the absolute increase in predictive ability from univariate GBLUP to maximum bivariate sERRBLUP on the respective sERRBLUP genomic correlation between 2017 and 2018 in Kemater (left) and in Petkuser (right) for all studied traits. In each panel, the overall linear regression line (gray solid line) with the regression coefficient (***b***) and R-squared (***R***^**2**^) are shown.

In addition to the comparison between the predictive abilities obtained by utilizing haplotype blocks versus pruned sets of SNPs, investigating genomic correlation as a factor affecting the bivariate models’ predictive abilities is of interest as bivariate models have found to outperform the univariate GBLUP in both haplotype blocks and pruned set of SNPs settings (Figs 1 and 2). The absolute gain in predictive ability from univariate GBLUP to maximum bivariate sERRBLUP, when utilizing haplotype blocks, was regressed on the respective sERRBLUP genomic correlation between the two respective years in each environment and across the series of studied traits (Fig 3). Regression coefficients range between -0.09and 0.81 and thus show a clear association between the absolute gain in prediction accuracy and the genomic correlation between the years in most environments. When combining all traits and environments, this correlation is 0.58 (p-value = 0.001) in KE and 0.66 (p-value = 0.0001) in PE. This correlation is also significant for most of the environments when utilizing pruned set of SNPs with the overall correlation of 0.65 (p-value = 0.00018) in KE and 0.69 (p-value = 4.393e-05) in PE (S17 Fig).

### Interplay of GBLUP and sERRBLUP prediction accuracy and genomic correlations

In order to investigate whether there is an underlying pattern when comparing the GBLUP genomic correlation and sERRBLUP genomic correlation leading to the maximum predictive ability, the genomic correlations across years estimated with GBLUP and sERRBLUP based on haplotype blocks and pruned set of SNPs for the trait PH_V4 are illustrated respectively in Tables 3 and 4. It is indicated that the proportion of interactions in bivariate sERRBLUP which maximized the predictive ability are not necessarily linked to the highest genomic correlation. In contrast, the best sERRBLUP for trait PH_V4 is linked to the lowest genomic correlation in most cases. However, this is not the general pattern observed for a series of other traits and the best sERRBLUP for some traits and environments combinations are linked to the highest genomic correlation based on both haplotype blocks (S7–S13 Tables) and pruned set of SNPs (S14–S20 Tables). In fact, there is a significant correlation between the absolute increase in predictive ability from bivariate GBLUP to maximum bivariate sERRBLUP and the difference between genetic correlations estimated with GBLUP and maximum sERRBLUP in both KE and PE when utilizing pruned set of SNPs (S18 Fig) and in KE when utilizing haplotype blocks (S19 Fig).

**Table 3:** Genomic correlation between 2017 and 2018 in each environment for trait PH_V4 for KE (blue numbers) and PE (red numbers). The blue and red bold numbers with stars indicate which proportion of interactions in bivariate sERRBLUP maximized the predictive ability based on haplotype blocks in each environment for KE and PE, respectively.

Bivariate Models	EIN	ROG	GOL	TOM
**GBLUP**	0.930/ 0.952	0. 984/ 0.712	0.930 / 0.959	0.927 / 0.911
**sERRBLUP top 10%**	0.940 / 0.882	0.954 / 0.649*	0.864 / 0.905	0.908 / 0.863*
**sERRBLUP top 5%**	0.0938 / 0.879	0.946 / 0.669	0.846 / 0.898	0.890 / 0.844
**sERRBLUP top 1%**	0.939 / 0.896*	0.937 / 0.833	0.825* / 0.893	0.860 / 0.858
**sERRBLUP top 0.1%**	0.943* / 0.902	0.939* / 0.898	0.873 / 0.916*	0.827* / 0.868
**sERRBLUP top 0.01%**	0.958 / 0.896	0.971 / 0.902	0.962 / 0.956	0.872 / 0.922
**sERRBLUP top 0.001%**	0.740 / 0.312	0.427 / 0.381	-0.998 / 0.505	0.515 / 0.525

**Table 4:** Genomic correlation between 2017 and 2018 in each environment for trait PH_V4 for KE (blue numbers) and PE (red numbers). The blue and red bold numbers with stars indicate which proportion of interactions in bivariate sERRBLUP maximized the predictive ability based on pruned set of SNPs in each environment for KE and PE, respectively.

Bivariate Models	EIN	ROG	GOL	TOM
**GBLUP**	0.945 / 0.898	0.940 / 0.658	0.942 / 0.969	0.954 / 0.923
**sERRBLUP top 10%**	0.955 / 0.859*	0.869* / 0.615*	0.835 / 0.895	0.929 / 0.816*
**sERRBLUP top 5%**	0.958 / 0.868	0.850 / 0.631	0.797 / 0.888	0.912 / 0.826
**sERRBLUP top 1%**	0.949 / 0.895	0.848 / 0.820	0.796* / 0.905*	0.918 / 0.863
**sERRBLUP top 0.1%**	0.962* / 0.966	0.917 / 0.922	0.884 / 0.948	0.929 / 0.959
**sERRBLUP top 0.01%**	0.963 / 0.980	0.951 / 0.985	0.911 / 0.983	0.919* / 0.987
**sERRBLUP top 0.001%**	0.997 / 0.976	0.963 / 0.970	0.908 / 0.973	0.933 / 0.968

### Correlation between prediction accuracy and the phenotypic correlation in bivariate models

**Fig 4:**
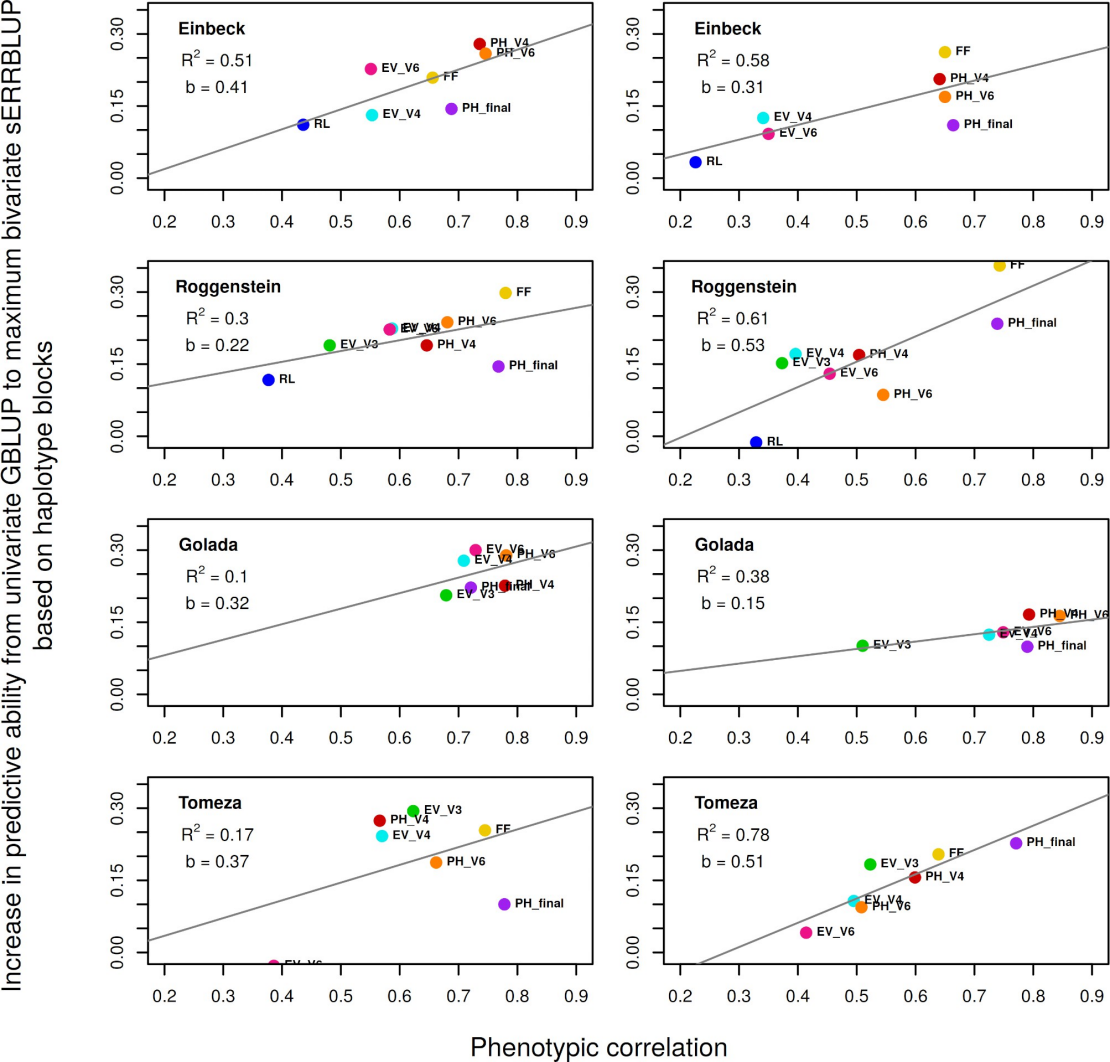
Regression of the absolute increase in predictive ability from univariate GBLUP to maximum bivariate sERRBLUP on the phenotypic correlation between 2017 and 2018 in Kemater (left) and in Petkuser (right) for all studied traits. In each panel, the overall linear regression line (gray solid line) with the regression coefficient (*b*) and R-squared (*R*^2^) are shown.

In addition to genomic correlation, phenotypic correlation can hypothetically have an impact on the bivariate models’ predictive ability, which is tested in this study. In fact, there might be a tendency that including phenotypes of the previous year into prediction becomes more efficient when the phenotypic correlation between years is high. In this context, the correlation between the absolute gain in predictive ability from univariate GBLUP to maximum bivariate sERRBLUP and the phenotypic correlation among the years (see S21 Table) over all studied traits in all four environments and in both landraces was studied. Fig 4 demonstrates that the maximum correlation between the absolute gain in the respective predictive ability based on the haplotype blocks and the phenotypic correlation is obtained in EIN for KE (0.51) and in TOM for PE (0.78). Across all studied traits and environments, there is a significant correlation of 0.55in KE (p-value = 0.002) and 0.56 in PE (p-value = 0.002). This correlation is also significant in most of the environments when utilizing pruned sets of SNPs with the overall correlation of 0.55 in KE (p-value = 0.003) and 0.50 in PE (p-value = 0.007) (S20 Fig).

### sERRBLUP computational load based on the pruned set of SNPs and haplotype blocks

As the obtained predictive abilities based on haplotype blocks is found to be almost identical to ones obtained based on pruned sets of SNPs (Figs 1 and 2), precise comparison on their computational load for sERRBLUP model as an epistasis model, which requires high computational load, is of great interest. In this regard, the required computational time for sERRBLUP based on 3’330 haplotype blocks with 5’546’115 interactions is found to be 9 minutes out of which 4 minutes are required to estimate the pairwise SNP interaction effect variances and 5 minutes are required to generate the sERRBLUP relationship matrix for a selected proportion of interactions by utilizing the R-package miraculix with 15 cores on a server cluster with Intel E5-2650 (2X12 core 2.2GHz) processors in the released EpiGP R-package [29]. As the computing time is increasing approximately quadratically with the number of included markers, the computing time for the respective SNP-based model with 30’212 SNPs takes 810 minutes, which is 90 times longer than haplotype block-based model [36], while it resulted in similar predictive abilities with the absolute difference being less than 0.01 in most cases across all traits in all environments and both landraces (S21 Fig).

## Discussion

In this study, we have found that GBLUP, ERRBLUP and maximum sERRBLUP predictive abilities when utilizing haplotype blocks are very similar to the respective models’ predictive abilities when utilizing pruned sets of SNPs. This finding is of high practical relevance, since it helps to overcome the high computational load of epistasis models as the computational time for haplotype block-based epistasis models is 90 times less than SNP-based epistasis models. Although we have found that the difference in predictive abilities is statistically significant based on a paired t-test, the relative gains from using sERRBLUP compared to traditional methods should massively outweigh these minor losses in accuracy, in particular, when testing the pipeline and performing cross validation schemes for parameter tuning (e.g. share of included interactions). In case computing time is not a problem the use of a larger data panel should still yield slightly better results.

Furthermore, the bivariate models for prediction across years behave in a similar manner as bivariate models for prediction across environments [36] such that ERRBLUP as a full epistasis model incorporating all pairwise SNP interactions is almost identical to bivariate GBLUP. This was expected, since ERRBLUP incorporates a high number of interactions by which a large number of unimportant variables are introduced into the model [6], thus introducing potential ‘noise’ which can prevent gains in predictive ability. In contrast, bivariate sERRBLUP substantially increases the predictive ability compared to bivariate GBLUP which is mainly caused by the inclusion of just the relevant pairwise SNP interactions. Note that all bivariate models substantially outperformed univariate GBLUP, as phenotypic data of the respective environment in the previous year was used, as was also observed for prediction across environments [36].

Although sERRBLUP is a method that is using multiple environments, it is not a GxE model [48] in the traditional sense. While GxE models typically assign effects to combinations of specific genotypes depending on the environment, the second environment in sERRBLUP is “only” used to detect which marker combinations affect a given trait to put more focus on these markers in the actual prediction step. The estimation of marker effects itself is then executed only based on the environment itself (or in the case of the bivariate model with some contributions from the second environment but still not in the sense of a traditional GxE model). As a different model is used for each environment, sERRBLUP will of course still assign different marker effects in different environments. As the set of selected marker interactions will be different between different models, a direct comparison of the effects assigned to specific marker interactions is not statistically sound. Similar to most GxE models [49], the computational load of sERRBLUP using markers is extremely high. However, our suggested use of haplotype blocks massively reduces this problem, while the predictive ability is almost as good as sERRBLUP based on the pruned set of SNPs.

It was shown that multivariate GBLUP is superior in predictive ability compared to univariate GBLUP with medium (~0.6) to high (~0.9) genomic correlations, and that low genomic correlations result in no increase in multivariate GBLUP compared to univariate GBLUP [11]. Calus *et al*. (2011) [50] also found an increase of 3 to 14 percent in predictive ability of multi-trait SNP-based models in a simulation study when genetic correlations ranged from 0.25 to 0.75. In our study, we also found a significant correlation between the absolute gain in prediction accuracy from univariate GBLUP to maximum bivariate sERRBLUP and the respective genomic correlation based on both pruned sets of SNPs (*r*_*KE*_ = 0.65, *r*_*PE*_ = 0.69) and haplotype blocks (*r*_*KE*_ = 0.47, *r*_*PE*_ = 0.49) across all traits and environments combinations.

Moreover, Martini et al. (2016) [6] showed that the predictive ability in one environment can be increased by variable selection in the other environment under the assumption of a positive phenotypic correlation between environments. It was shown in a wheat dataset [51], where environments 2 and 3 had the highest phenotypic correlation (0.661), that the predictive ability for phenotype prediction in environment 2 was maximized by variable selection in environment 3 and vice versa [6]. Although 2017 and 2018 were climatically quite different, since 2018 suffered from major heat stress compared to 2017 (Table 1), we still see a significant correlation between the absolute gain in predictive ability from univariate GBLUP to maximum predictive ability of bivariate sERRBLUP and the phenotypic correlation between years in each environment based on both pruned sets of SNPs (*r*_*KE*_ = 0.55, *r*_*PE*_ = 0.50) and haplotype blocks (*r*_*KE*_ = 0.55, *r*_*PE*_ = 0.56).

In addition to the genomic and phenotypic correlations between the years, the trait heritability is another factor that is expected to be influential for such an increase in bivariate sERRBLUP predictive ability as well. Therefore, the traits with lower heritability are expected to obtain less gain in sERRBLUP predictive ability than the traits with higher heritability. This was confirmed in our study, as traits with low heritability (e.g. 0.59 for RL in PE) showed only a small increase in prediction accuracy from univariate GBLUP to maximum bivariate sERRBLUP. However, not all traits with higher heritabilities did necessarily show a higher gain in predictive ability for all traits. It should be noted that the trait heritabilities were calculated on an entry-mean basis [14] within each KE and PE landraces by [37] over all four environments in both years 2017 and 2018 jointly. The trait heritabilities obtained only from 2017 are significantly higher than the trait heritabilities obtained only from 2018 in both KE and PE based on a paired t-test (S2 Table). This partly explains the increase in predictive ability from univariate GBLUP to maximum bivariate sERRBLUP in KE and PE, since multi-trait models have the potential of increasing the predictive ability when traits with low heritability are joined with traits with higher heritability, given they are genomically correlated [52].

It should be noted that the increase in predictive ability from univariate GBLUP to maximum bivariate sERRBLUP is caused by both borrowing information across years and capitalizing on epistasis, while the increase in predictive ability from bivariate GBLUP to maximum bivariate sERRBLUP is caused by accounting for epistasis only. Overall, the traits behave differently among different environments and landraces due to their genomic correlations, phenotypic correlations and heritabilities. To shed light on this, the maximum increase in prediction accuracy from bivariate GBLUP to bivariate sERRBLUP based on the pruned set of SNPs in KE was observed for the trait EV_V6 (0.112) in EIN where the corresponding sERRBLUP genomic correlation (0.809) is higher than the GBLUP genomic correlation (0.768). This trait has a high heritability (0.90) and high phenotypic correlation (0.551) as well. In contrast, the respective prediction accuracy decreases (-0.018) for EV_V6 in TOM for KE indicating the lower sERRBLUP genomic correlation (0.458) than GBLUP genomic correlation (0.703) and the particularly low phenotypic correlation (0.383). It should be noted that the phenotypic correlation does not play a major role for the increase in prediction accuracy from bivariate GBLUP to bivariate sERRBLUP, since both models are bivariate and benefit from the same phenotypic correlations. Therefore, EV_V6 obtaining the maximum and minimum increase in the respective prediction accuracy for KE indicates the significant role of genomic correlation among the possible causes. In general, bivariate sERRBLUP improves the prediction accuracy compared to bivariate GBLUP more in KE than PE which is potentially due to significantly higher sERRBLUP genomic correlation and heritability in KE compared to PE, based on paired t-test.

Overall, we conclude that utilizing haplotype blocks instead of pruned sets of SNPs in epistasis models helps to makes ERRBLUP practically applicable, since the obtained predictive abilities of the best epistasis model are quite similar, while the required computational time when utilizing haplotype blocks is massively lower compared to using pruned sets of SNPs. Moreover, our results indicate that incorporating a suitable subset of epistatic interactions besides utilizing information across years can substantially increase the predictive ability. The amount of this increase is affected by the genomic and phenotypic correlations between the years and the heritability of the phenotypic trait. The suggested approach is especially beneficial for genomic prediction of phenotypes under the assumption of sufficient genomic and phenotypic correlation between years for highly heritable traits. This may allow to reduce the number of lines that have to be phenotyped over several years and thus to reduce phenotyping costs which may be of high interest in practical plant breeding.

## Supporting information

S1 Fig Comparison of pre-estimated genetic and residual variances and covariances of converged bivariate sERRBLUP (top 10%) based on the full dataset (dashed horizontal lines) and estimated genetic and residual variances and covariances of converged bivariate sERRBLUP (top 10%) based on training set in each run of 5-fold cross validation with 5 replicates (colored bars) for predicting EIN in 2018 when the additional environment is EIN in 2017 in KE for trait PH-V4. (DOCX)

S2 Fig The difference between the mean predictive ability of only the converged folds and the mean predictive ability of all folds in 5-fold cross validation with 5 replicates virus the number of the folds which did not converged across all traits in all combinations for both KE and PE in bivariate GBLUP, ERRBLUP, sERRBLUP. (DOCX)

S3 Fig Predictive ability for univariate GBLUP within 2018 (orange and red dashed horizontal line), bivariate GBLUP (green and blue dashed horizontal line), bivariate ERRBLUP (open circle) and bivariate sERRBLUP (filled circles and solid line) for trait EV_V3 in KE based on Pruned set of SNPs (left) and haplotype blocks (right). In each plot, the sERRBLUP maximum indicates the maximum predictive ability obtained from bivariate sERRBLUP.
(DOCX)

S4 Fig Predictive ability for univariate GBLUP within 2018 (orange and red dashed horizontal line), bivariate GBLUP (green and blue dashed horizontal line), bivariate ERRBLUP (open circle) and bivariate sERRBLUP (filled circles and solid line) for trait EV_V3 in PE based on Pruned set of SNPs (left) and haplotype blocks (right). In each plot, the sERRBLUP maximum indicates the maximum predictive ability obtained from bivariate sERRBLUP.
(DOCX)

S5 Fig Predictive ability for univariate GBLUP within 2018 (orange and red dashed horizontal line), bivariate GBLUP (green and blue dashed horizontal line), bivariate ERRBLUP (open circle) and bivariate sERRBLUP (filled circles and solid line) for trait EV_V4 in KE based on Pruned set of SNPs (left) and haplotype blocks (right). In each plot, the sERRBLUP maximum indicates the maximum predictive ability obtained from bivariate sERRBLUP.
(DOCX)

S6 Fig Predictive ability for univariate GBLUP within 2018 (orange and red dashed horizontal line), bivariate GBLUP (green and blue dashed horizontal line), bivariate ERRBLUP (open circle) and bivariate sERRBLUP (filled circles and solid line) for trait EV_V4 in PE based on Pruned set of SNPs (left) and haplotype blocks (right). In each plot, the sERRBLUP maximum indicates the maximum predictive ability obtained from bivariate sERRBLUP.
(DOCX)

S7 Fig Predictive ability for univariate GBLUP within 2018 (orange and red dashed horizontal line), bivariate GBLUP (green and blue dashed horizontal line), bivariate ERRBLUP (open circle) and bivariate sERRBLUP (filled circles and solid line) for trait EV_V6 in KE based on Pruned set of SNPs (left) and haplotype blocks (right). In each plot, the sERRBLUP maximum indicates the maximum predictive ability obtained from bivariate sERRBLUP.
(DOCX)

S8 Fig Predictive ability for univariate GBLUP within 2018 (orange and red dashed horizontal line), bivariate GBLUP (green and blue dashed horizontal line), bivariate ERRBLUP (open circle) and bivariate sERRBLUP (filled circles and solid line) for trait EV_V6 in PE based on Pruned set of SNPs (left) and haplotype blocks (right). In each plot, the sERRBLUP maximum indicates the maximum predictive ability obtained from bivariate sERRBLUP.
(DOCX)

S9 Fig Predictive ability for univariate GBLUP within 2018 (orange and red dashed horizontal line), bivariate GBLUP (green and blue dashed horizontal line), bivariate ERRBLUP (open circle) and bivariate sERRBLUP (filled circles and solid line) for trait PH_V6 in KE based on Pruned set of SNPs (left) and haplotype blocks (right). In each plot, the sERRBLUP maximum indicates the maximum predictive ability obtained from bivariate sERRBLUP.
(DOCX)

S10 Fig Predictive ability for univariate GBLUP within 2018 (orange and red dashed horizontal line), bivariate GBLUP (green and blue dashed horizontal line), bivariate ERRBLUP (open circle) and bivariate sERRBLUP (filled circles and solid line) for trait PH_V6 in PE based on Pruned set of SNPs (left) and haplotype blocks (right). In each plot, the sERRBLUP maximum indicates the maximum predictive ability obtained from bivariate sERRBLUP.
(DOCX)

S11 Fig Predictive ability for univariate GBLUP within 2018 (orange and red dashed horizontal line), bivariate GBLUP (green and blue dashed horizontal line), bivariate ERRBLUP (open circle) and bivariate sERRBLUP (filled circles and solid line) for trait PH_final in KE based on Pruned set of SNPs (left) and haplotype blocks (right). In each plot, the sERRBLUP maximum indicates the maximum predictive ability obtained from bivariate sERRBLUP.
(DOCX)

S12 Fig Predictive ability for univariate GBLUP within 2018 (orange and red dashed horizontal line), bivariate GBLUP (green and blue dashed horizontal line), bivariate ERRBLUP (open circle) and bivariate sERRBLUP (filled circles and solid line) for trait PH_final in PE based on Pruned set of SNPs (left) and haplotype blocks (right). In each plot, the sERRBLUP maximum indicates the maximum predictive ability obtained from bivariate sERRBLUP.
(DOCX)

S13 Fig Predictive ability for univariate GBLUP within 2018 (orange and red dashed horizontal line), bivariate GBLUP (green and blue dashed horizontal line), bivariate ERRBLUP (open circle) and bivariate sERRBLUP (filled circles and solid line) for trait FF in KE based on Pruned set of SNPs (left) and haplotype blocks (right). In each plot, the sERRBLUP maximum indicates the maximum predictive ability obtained from bivariate sERRBLUP.
(DOCX)

S14 Fig Predictive ability for univariate GBLUP within 2018 (orange and red dashed horizontal line), bivariate GBLUP (green and blue dashed horizontal line), bivariate ERRBLUP (open circle) and bivariate sERRBLUP (filled circles and solid line) for trait FF in PE based on Pruned set of SNPs (left) and haplotype blocks (right). In each plot, the sERRBLUP maximum indicates the maximum predictive ability obtained from bivariate sERRBLUP.
(DOCX)

S15 Fig Predictive ability for univariate GBLUP within 2018 (orange and red dashed horizontal line), bivariate GBLUP (green and blue dashed horizontal line), bivariate ERRBLUP (open circle) and bivariate sERRBLUP (filled circles and solid line) for trait RL in KE based on Pruned set of SNPs (left) and haplotype blocks (right). In each plot, the sERRBLUP maximum indicates the maximum predictive ability obtained from bivariate sERRBLUP.
(DOCX)

S16 Fig Predictive ability for univariate GBLUP within 2018 (orange and red dashed horizontal line), bivariate GBLUP (green and blue dashed horizontal line), bivariate ERRBLUP (open circle) and bivariate sERRBLUP (filled circles and solid line) for trait RL in PE based on Pruned set of SNPs (left) and haplotype blocks (right). In each plot, the sERRBLUP maximum indicates the maximum predictive ability obtained from bivariate sERRBLUP.
(DOCX)

S17 Fig Regression of the absolute increase in predictive ability from univariate GBLUP to maximum bivariate sERRBLUP on the respective sERRBLUP genomic correlation between 2017 and 2018 in KE (left) and in PE (right) for all studied traits. In each panel, the overall linear regression line (gray solid line) with the regression coefficient (***b***) and R-squared (***R***^**2**^) are shown.
(DOCX)

S18 Fig Regression of the absolute increase in predictive ability from bivariate GBLUP to maximum bivariate sERRBLUP on the difference between the GBLUP genomic correlation and maximum sERRBLUP genomic correlation between 2017 and 2018 in KE (left) and in PE (right) for all studied traits, when utilizing pruned set of SNPs. In each panel, the overall linear regression line with the regression coefficient (***b***) and R-squared (***R***^**2**^) are shown. The colors green, light blue, pink, red, orange, purple, yellow and dark blue represent the phenotypic traits EV_V3, EV_V4, EV_V6, PH_V4, PH_V6, PH_final, FF and RL, respectively.
(DOCX)

S19 Fig Regression of the absolute increase in predictive ability from bivariate GBLUP to maximum bivariate sERRBLUP on the difference between the GBLUP genomic correlation and maximum sERRBLUP genomic correlation between 2017 and 2018 in KE (left) and in PE (right) for all studied traits, when utilizing haplotype blocks. In each panel, the overall linear regression line with the regression coefficient (***b***) and R-squared (***R***^**2**^) are shown. The colors green, light blue, pink, red, orange, purple, yellow and dark blue represent the phenotypic traits EV_V3, EV_V4, EV_V6, PH_V4, PH_V6, PH_final, FF and RL, respectively.
(DOCX)

S20 Fig Regression of the absolute increase in predictive ability from univariate GBLUP to maximum bivariate sERRBLUP on the phenotypic correlation between 2017 and 2018 in KE (left) and in PE (right) for all studied traits. In each panel, the overall linear regression line (gray solid line) with the regression coefficient (***b***) and R-squared (***R***^**2**^) are shown.
(DOCX)

S21 Fig Maximum sERRBLUP predictive ability based on pruned set of SNPs versus Maximum sERRBLUP predictive ability based on haplotype blocks across all traits in all environments and both KE and PE. *b* represents the regression coefficient.
(DOCX)

S1 Table The mean, minimum, maximum and standard deviation of BLUEs of phenotypic traits in each location for KE (blue numbers) and PE (red numbers) in 2017 and 2018. (DOCX)

S2 Table The traits heritabilities in 2017, 2018 and both years jointly in KE (blue numbers) and PE (red numbers). (DOCX)

S3 Table The percentage of bivariate models converged in 5-fold cross validation with 5 replicates based on pruned set of SNPs for both KE and PE (black percentages), only KE (blue percentages) and only PE (red percentages). The stars represent the non-convergence of pre-estimated variance components based on the full set.
(DOCX)

S4 Table The number of epistasis interactions maintained in the model based on haplotype blocks and pruned set of SNPs for each proportions of interactions in KE and PE. (DOCX)

S5 Table GBLUP predictive ability based on pruned set of SNPs for prediction in 2018 with training the model either on 2018 data or the average phenotypic values of 2017 and 2018 in each environment for series of phenotypic traits in KE. (DOCX)

S6 Table GBLUP predictive ability based on pruned set of SNPs for prediction in 2018 with training the model either on 2018 data or the average phenotypic values of 2017 and 2018 in each environment for series of phenotypic traits in PE. (DOCX)

S7 Table Genomic correlation between 2017 and 2018 in each environment for trait EV_V3 for KE (blue numbers) and PE (red numbers). The blue and red bold numbers with stars indicate which proportion of interactions in bivariate sERRBLUP maximized the predictive ability based on haplotype blocks in each environment for KE and PE, respectively.
(DOCX)

S8 Table Genomic correlation between 2017 and 2018 in each environment for trait EV_V4 for KE (blue numbers) and PE (red numbers). The blue and red bold numbers with stars indicate which proportion of interactions in bivariate sERRBLUP maximized the predictive ability based on haplotype blocks in each environment for KE and PE, respectively.
(DOCX)

S9 Table Genomic correlation between 2017 and 2018 in each environment for trait EV_V6 for KE (blue numbers) and PE (red numbers). The blue and red bold numbers with stars indicate which proportion of interactions in bivariate sERRBLUP maximized the predictive ability based on haplotype blocks in each environment for KE and PE, respectively.
(DOCX)

S10 Table Genomic correlation between 2017 and 2018 in each environment for trait PH_V6 for KE (blue numbers) and PE (red numbers). The blue and red bold numbers with stars indicate which proportion of interactions in bivariate sERRBLUP maximized the predictive ability based on haplotype blocks in each environment for KE and PE, respectively.
(DOCX)

S11 Table Genomic correlation between 2017 and 2018 in each environment for trait PH_final for KE (blue numbers) and PE (red numbers). The blue and red bold numbers with stars indicate which proportion of interactions in bivariate sERRBLUP maximized the predictive ability based on haplotype blocks in each environment for KE and PE, respectively.
(DOCX)

S12 Table Genomic correlation between 2017 and 2018 in each environment for trait FF for KE (blue numbers) and PE (red numbers). The blue and red bold numbers with stars indicate which proportion of interactions in bivariate sERRBLUP maximized the predictive ability based on haplotype blocks in each environment for KE and PE, respectively.
(DOCX)

S13 Table Genomic correlation between 2017 and 2018 in each environment for trait RL for KE (blue numbers) and PE (red numbers). The blue and red bold numbers with stars indicate which proportion of interactions in bivariate sERRBLUP maximized the predictive ability based on haplotype blocks in each environment for KE and PE, respectively.
(DOCX)

S14 Table Genomic correlation between 2017 and 2018 in each environment for trait EV_V3 for KE (blue numbers) and PE (red numbers). The blue and red bold numbers with stars indicate which proportion of interactions in bivariate sERRBLUP maximized the predictive ability based on pruned set of SNPs in each environment for KE and PE, respectively.
(DOCX)

S15 Table Genomic correlation between 2017 and 2018 in each environment for trait EV_V4 for KE (blue numbers) and PE (red numbers). The blue and red bold numbers with stars indicate which proportion of interactions in bivariate sERRBLUP maximized the predictive ability based on pruned set of SNPs in each environment for KE and PE, respectively.
(DOCX)

S16 Table Genomic correlation between 2017 and 2018 in each environment for trait EV_V6 for KE (blue numbers) and PE (red numbers). The blue and red bold numbers with stars indicate which proportion of interactions in bivariate sERRBLUP maximized the predictive ability based on pruned set of SNPs in each environment for KE and PE, respectively.
(DOCX)

S17 Table Genomic correlation between 2017 and 2018 in each environment for trait PH_V6 for KE (blue numbers) and PE (red numbers). The blue and red bold numbers with stars indicate which proportion of interactions in bivariate sERRBLUP maximized the predictive ability based on pruned set of SNPs in each environment for KE and PE, respectively.
(DOCX)

S18 Table Genomic correlation between 2017 and 2018 in each environment for trait PH_final for KE (blue numbers) and PE (red numbers). The blue and red bold numbers with stars indicate which proportion of interactions in bivariate sERRBLUP maximized the predictive ability based on pruned set of SNPs in each environment for KE and PE, respectively.
(DOCX)

S19 Table Genomic correlation between 2017 and 2018 in each environment for trait FF for KE (blue numbers) and PE (red numbers). The blue and red bold numbers with stars indicate which proportion of interactions in bivariate sERRBLUP maximized the predictive ability based on pruned set of SNPs in each environment for KE and PE, respectively.
(DOCX)

S20 Table Genomic correlation between 2017 and 2018 in each environment for trait RL for KE (blue numbers) and PE (red numbers). The blue and red bold numbers with stars indicate which proportion of interactions in bivariate sERRBLUP maximized the predictive ability based on pruned set of SNPs in each environment for KE and PE, respectively.
(DOCX)

S21 Table Phenotypic correlation between 2017 and 2018 in each environment for KE and PE. (DOCX)
