# Supplementary material for: Genomic prediction using information across years with epistatic models and dimension reduction via haplotype blocks
Source: PLoS One. 2023 Mar 31;18(3):e0282288. doi: 10.1371/journal.pone.0282288 (PMC10065328; doi:10.1371/journal.pone.0282288)
Supplement: S1 Fig — (DOCX) [file pone.0282288.s001.docx]

**Supplemental Figures**

**
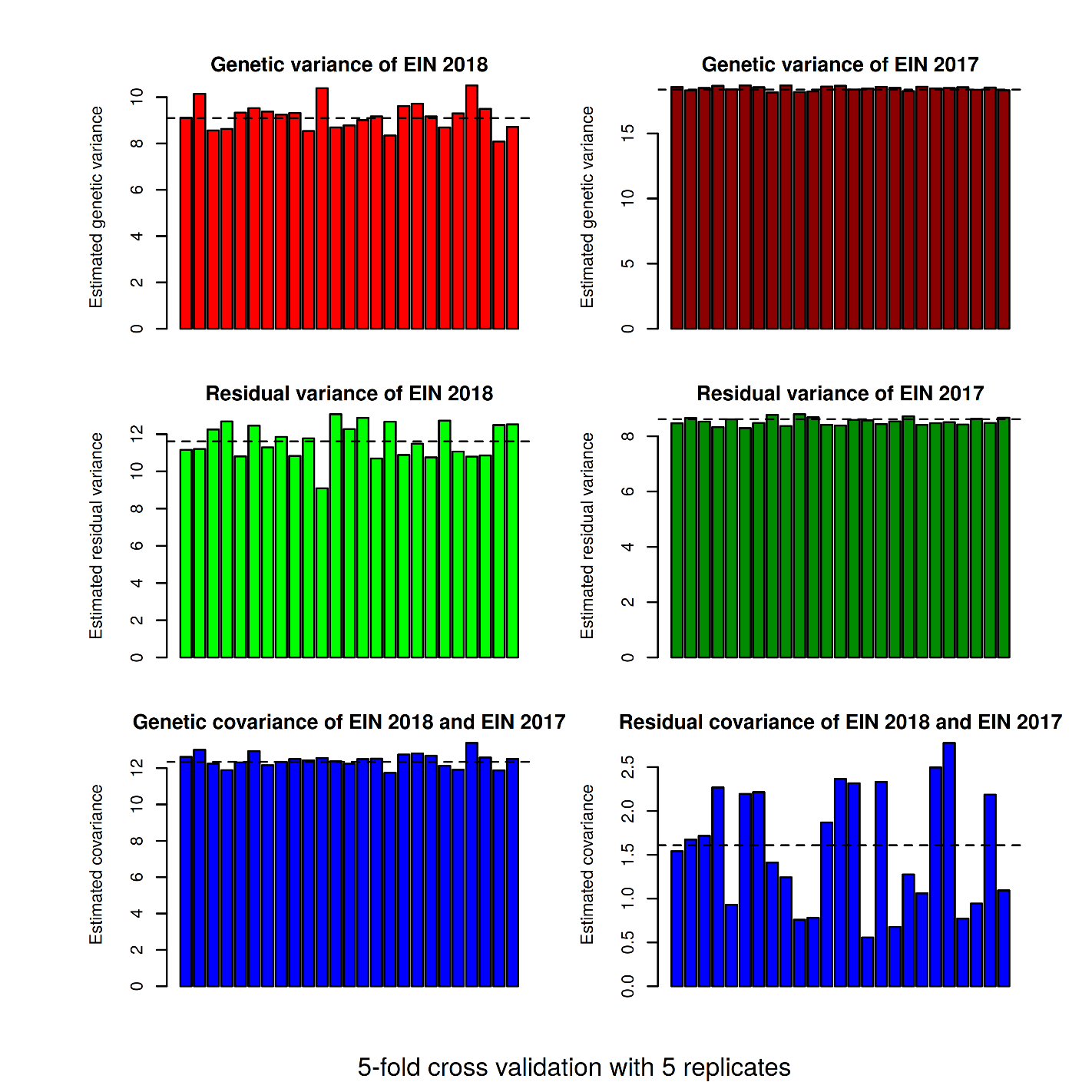
**

**S1** **Fig.** Comparison of pre-estimated genetic and residual variances and covariances of converged bivariate sERRBLUP (top 10%) based on the full dataset (dashed horizontal lines) and estimated genetic and residual variances and covariances of converged bivariate sERRBLUP (top 10%) based on training set in each run of 5-fold cross validation with 5 replicates (colored bars) for predicting EIN in 2018 when the additional environment is EIN in 2017 in KE for trait PH-V4.
