# Supplementary material for: Genomic prediction using information across years with epistatic models and dimension reduction via haplotype blocks
Source: PLoS One. 2023 Mar 31;18(3):e0282288. doi: 10.1371/journal.pone.0282288 (PMC10065328; doi:10.1371/journal.pone.0282288)
Supplement: S2 Fig — (DOCX) [file pone.0282288.s002.docx]

**
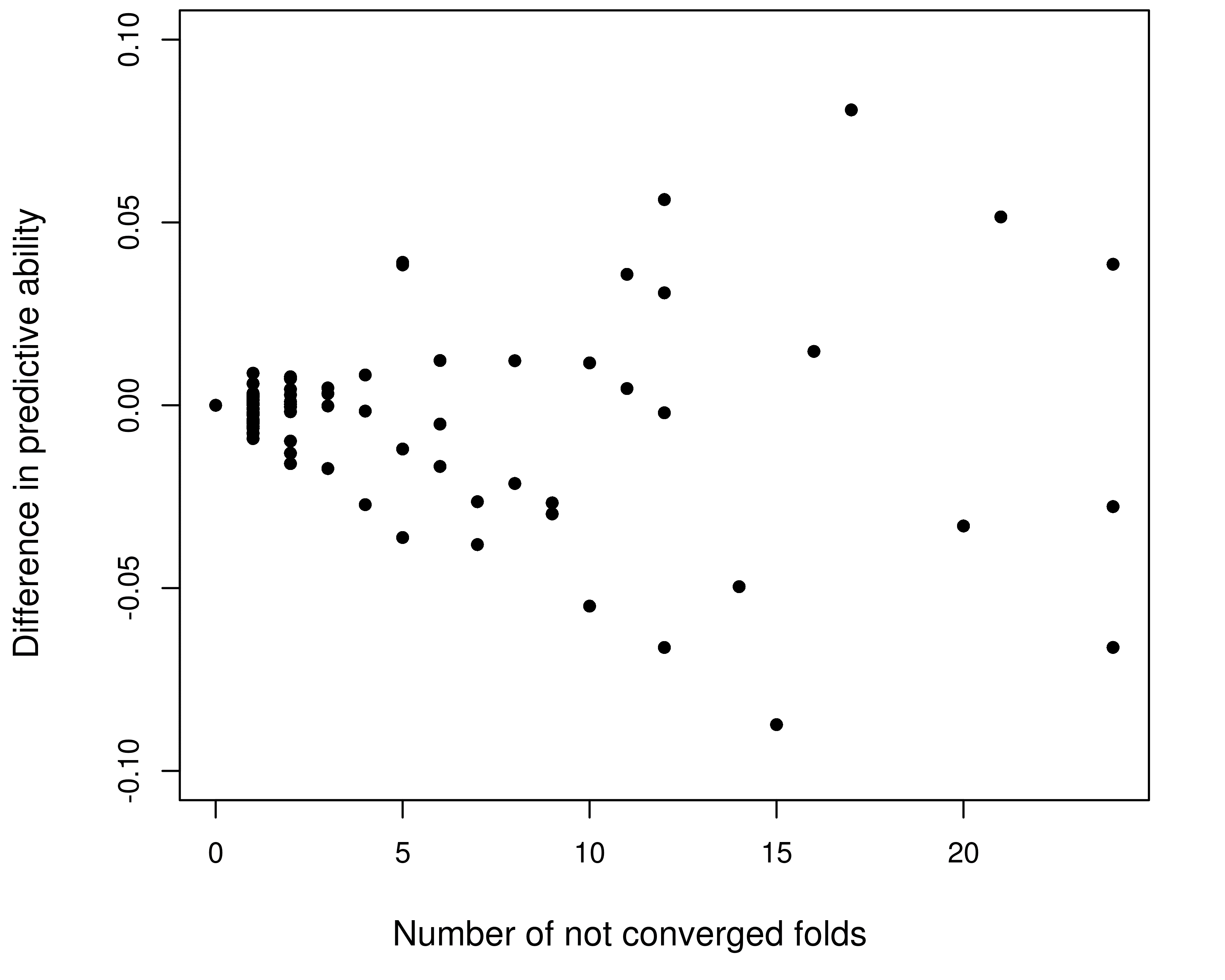
**

**S2** **Fig.** The difference between the mean predictive ability of only the converged folds and the mean predictive ability of all folds in 5-fold cross validation with 5 replicates virus the number of the folds which did not converged across all traits in all combinations for both KE and PE in bivariate GBLUP, ERRBLUP, sERRBLUP.
