# Supplementary material for: Genomic prediction using information across years with epistatic models and dimension reduction via haplotype blocks
Source: PLoS One. 2023 Mar 31;18(3):e0282288. doi: 10.1371/journal.pone.0282288 (PMC10065328; doi:10.1371/journal.pone.0282288)
Supplement: S4 Fig — Predictive ability for univariate GBLUP within 2018 (orange and red dashed horizontal line), bivariate GBLUP (green and blue dashed horizontal line), bivariate ERRBLUP (open circle) and bivariate sERRBLUP (filled circles and solid line) for trait EV_V3 in PE based on Pruned set of SNPs (left) and haplotype blocks (right). In each plot, the sERRBLUP maximum indicates the maximum predictive ability obtained from bivariate sERRBLUP. (DOCX) [file pone.0282288.s004.docx]

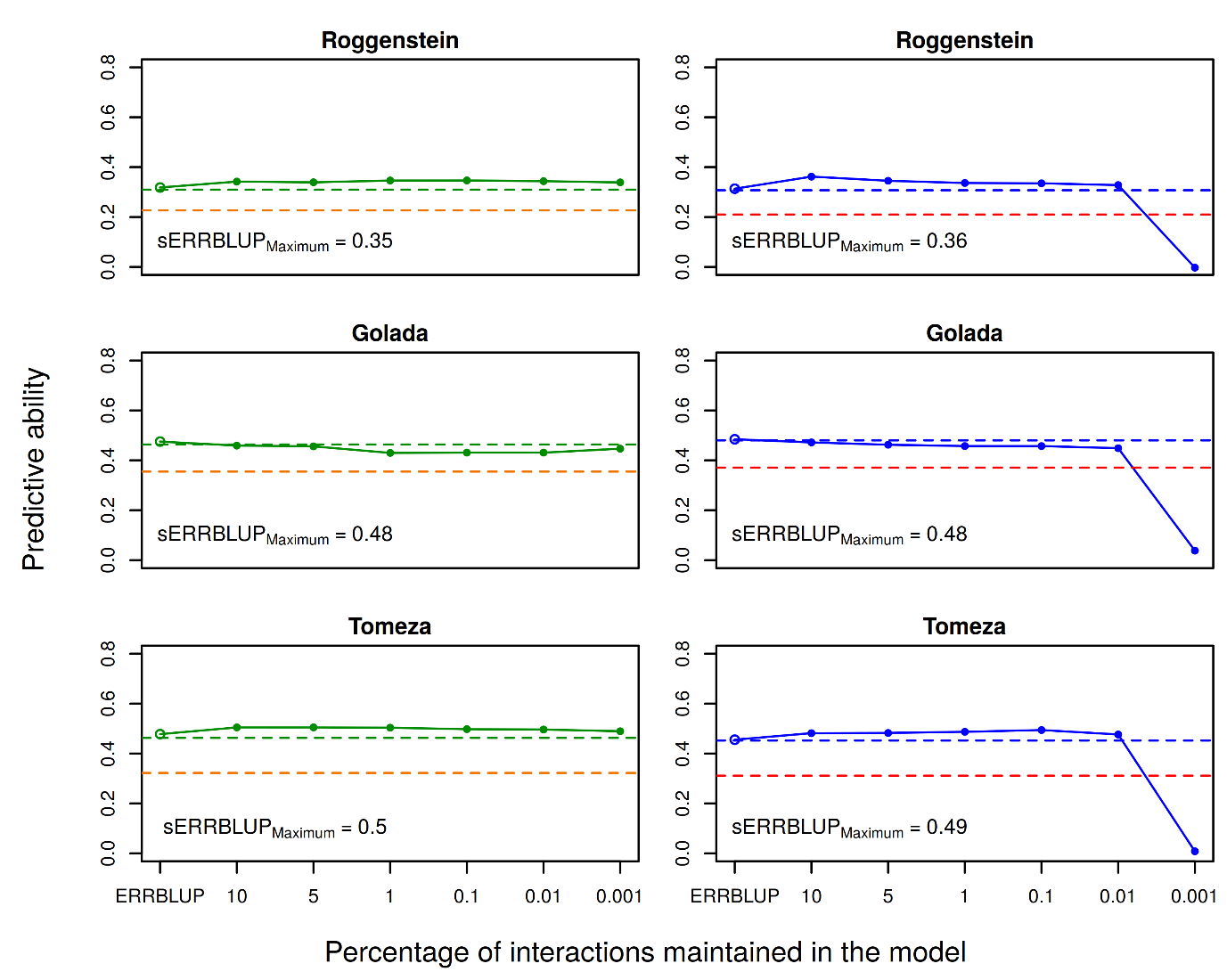


**S4** **Fig.** Predictive ability for univariate GBLUP within 2018 (orange and red dashed horizontal line), bivariate GBLUP (green and blue dashed horizontal line), bivariate ERRBLUP (open circle) and bivariate sERRBLUP (filled circles and solid line) for trait EV_V3 in PE based on Pruned set of SNPs (left) and haplotype blocks (right). In each plot, the sERRBLUP maximum indicates the maximum predictive ability obtained from bivariate sERRBLUP.
