# Supplementary material for: Genomic prediction using information across years with epistatic models and dimension reduction via haplotype blocks
Source: PLoS One. 2023 Mar 31;18(3):e0282288. doi: 10.1371/journal.pone.0282288 (PMC10065328; doi:10.1371/journal.pone.0282288)
Supplement: S17 Fig — Regression of the absolute increase in predictive ability from univariate GBLUP to maximum bivariate sERRBLUP on the respective sERRBLUP genomic correlation between 2017 and 2018 in KE (left) and in PE (right) for all studied traits. In each panel, the overall linear regression line (gray solid line) with the regression coefficient (b) and R-squared (R2) are shown. (DOCX) [file pone.0282288.s017.docx]

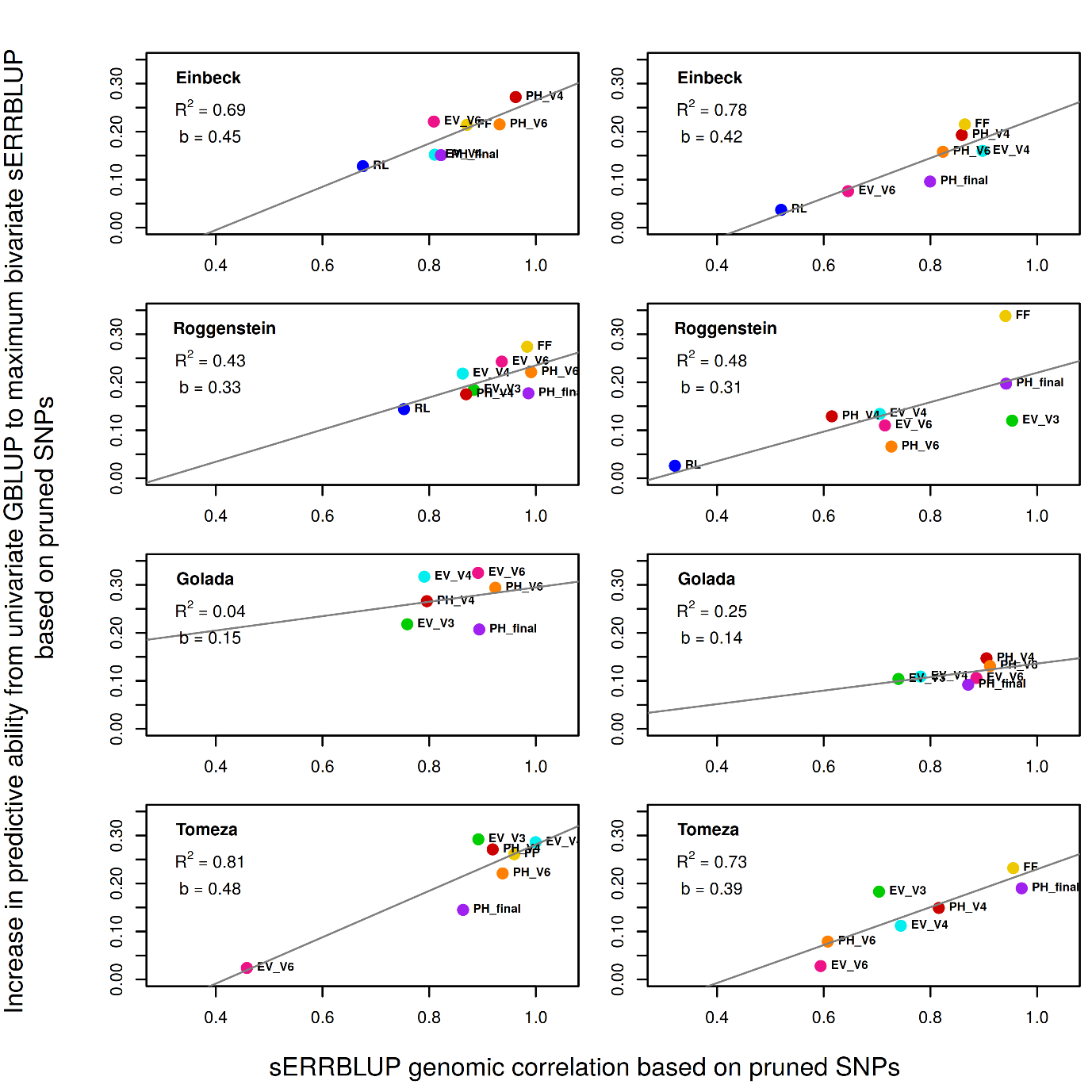


**S17 Fig.** Regression of the absolute increase in predictive ability from univariate GBLUP to maximum bivariate sERRBLUP on the respective sERRBLUP genomic correlation between 2017 and 2018 in KE (left) and in PE (right) for all studied traits. In each panel, the overall linear regression line (gray solid line) with the regression coefficient ($\boldsymbol{b}$) and R-squared ($\boldsymbol{R}^{\boldsymbol{2}}$) are shown.
