# Supplementary material for: Genomic prediction using information across years with epistatic models and dimension reduction via haplotype blocks
Source: PLoS One. 2023 Mar 31;18(3):e0282288. doi: 10.1371/journal.pone.0282288 (PMC10065328; doi:10.1371/journal.pone.0282288)
Supplement: S18 Fig — Regression of the absolute increase in predictive ability from bivariate GBLUP to maximum bivariate sERRBLUP on the difference between the GBLUP genomic correlation and maximum sERRBLUP genomic correlation between 2017 and 2018 in KE (left) and in PE (right) for all studied traits, when utilizing pruned set of SNPs. In each panel, the overall linear regression line with the regression coefficient (b) and R-squared (R2) are shown. The colors green, light blue, pink, red, orange, purple, yellow and dark blue represent the phenotypic traits EV_V3, EV_V4, EV_V6, PH_V4, PH_V6, PH_final, FF and RL, respectively. (DOCX) [file pone.0282288.s018.docx]

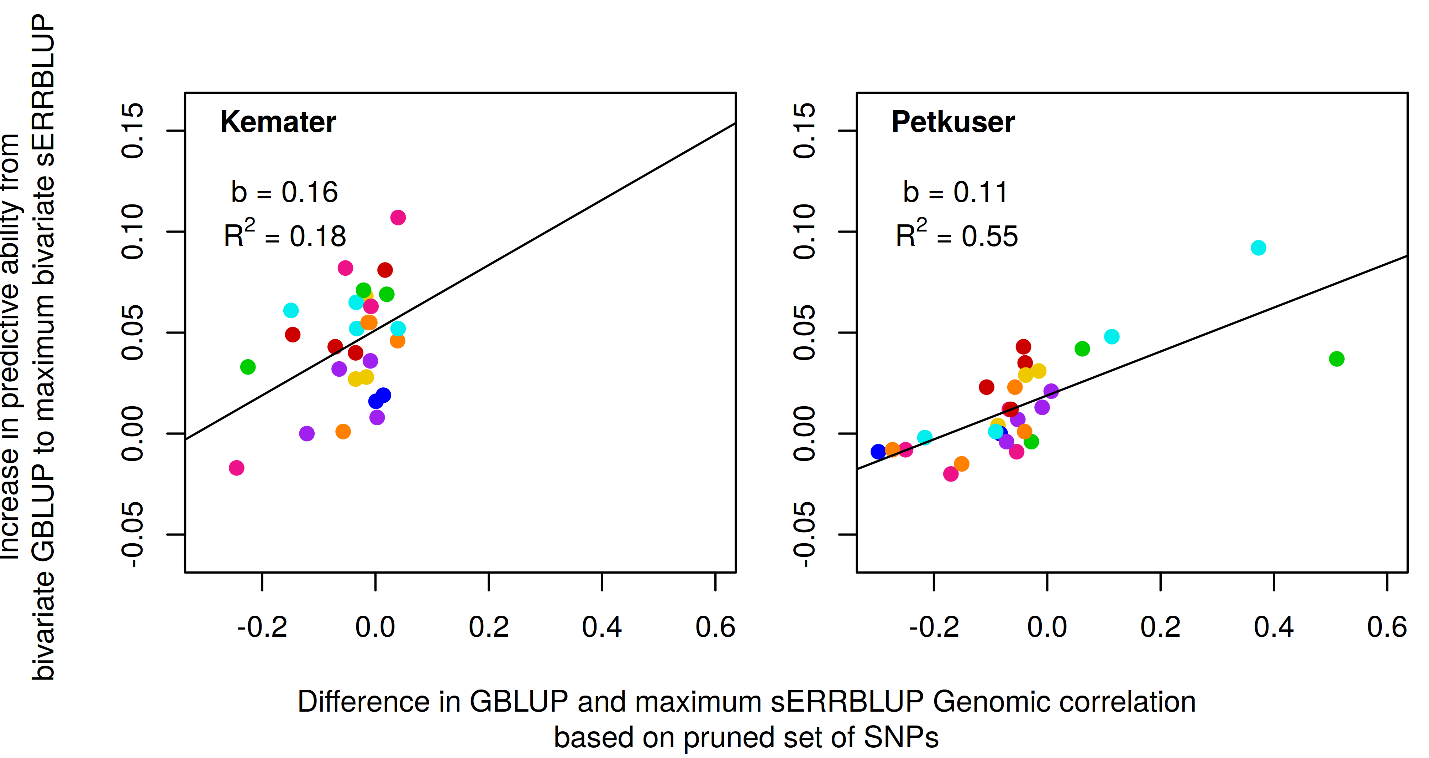


**S18 Fig.** Regression of the absolute increase in predictive ability from bivariate GBLUP to maximum bivariate sERRBLUP on the difference between the GBLUP genomic correlation and maximum sERRBLUP genomic correlation between 2017 and 2018 in KE (left) and in PE (right) for all studied traits, when utilizing pruned set of SNPs. In each panel, the overall linear regression line with the regression coefficient ($\boldsymbol{b}$) and R-squared ($\boldsymbol{R}^{\boldsymbol{2}}$) are shown. The colors green, light blue, pink, red, orange, purple, yellow and dark blue represent the phenotypic traits EV_V3, EV_V4, EV_V6, PH_V4, PH_V6, PH_final, FF and RL, respectively.
