# Supplementary material for: Genomic prediction using information across years with epistatic models and dimension reduction via haplotype blocks
Source: PLoS One. 2023 Mar 31;18(3):e0282288. doi: 10.1371/journal.pone.0282288 (PMC10065328; doi:10.1371/journal.pone.0282288)
Supplement: S21 Fig — b represents the regression coefficient. (DOCX) [file pone.0282288.s021.docx]

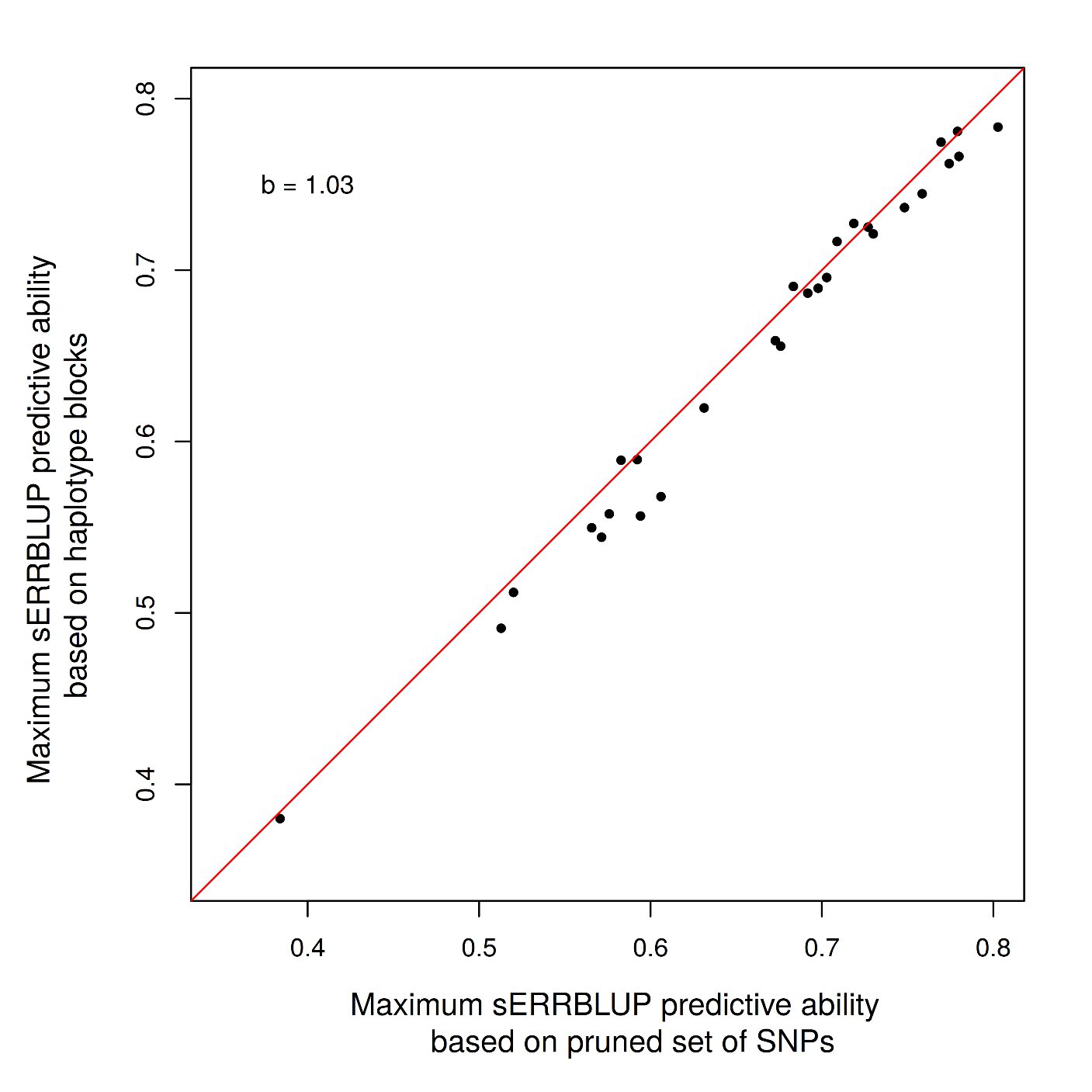


**S21 Fig.** Maximum sERRBLUP predictive ability based on pruned set of SNPs versus Maximum sERRBLUP predictive ability based on haplotype blocks across all traits in all environments and both KE and PE. $\boldsymbol{b}$ represents the regression coefficient.
