# Supplementary material for: Genomic prediction using information across years with epistatic models and dimension reduction via haplotype blocks
Source: PLoS One. 2023 Mar 31;18(3):e0282288. doi: 10.1371/journal.pone.0282288 (PMC10065328; doi:10.1371/journal.pone.0282288)
Supplement: S1 Table — (DOCX) [file pone.0282288.s022.docx]

**Genomic prediction using information across years with epistatic models and dimension reduction via haplotype blocks**

**Elaheh Vojgani^1*^, Armin C. Hölker^2^, Manfred Mayer^2^, Chris-Carolin Schön^2^, Henner Simianer^3^ Torsten Pook^3^**

^1^Cologne Center for Genomics, University of Cologne, Cologne, Germany; ^2^Plant Breeding, TUM School of Life Sciences Weihenstephan, Technical University of Munich, Freising, Germany; ^3^Center for Integrated Breeding Research, Animal Breeding and Genetics Group, University of Goettingen, Goettingen, Germany

**Corresponding author**

Correspondence to Elaheh Vojgani

Email: [vojgani@gwdg.de](mailto:vojgani@gwdg.de)

ORCID: 0000-0003-4375-3531

**Supplemental Tables**

**S1** **Table.** The mean, minimum, maximum and standard deviation of BLUEs of phenotypic traits in each location for KE (blue numbers) and PE (red numbers) in 2017 and 2018.

| Trait | Location/Year | Mean | Minimum | Maximum | Standard deviation |
| --- | --- | --- | --- | --- | --- |
| EV_V3 | **ROG/2017**  **ROG/2018**  **GOL/2017**  **GOL/2018**  **TOM/2017**  **TOM/2018** | 5.35\5.84  5.68\5.91  6.31\6.67  4.26\4.62  5.51\6.15  4.84\5.67 | 1.71\2.90  1.21\3.46  4.07\5.49  0.32\1.69  1.93\3.84  0.59\1.38 | 7.90\7.92  7.91\8.08  8.49\7.98  7.63\7.99  7.34\8.45  8.67\8.93 | 0.95\0.75  1.01\0.89  0.69\0.51  1.20\0.97  0.99\0.67  1.43\1.13 |
| EV_V4 | **EIN/2017**  **EIN/2018**  **ROG/2017**  **ROG/2018**  **GOL/2017**  **GOL/2018**  **TOM/2017**  **TOM/2018** | 4.24\4.82  4.56\4.61  5.44\5.85  5.61\5.84  5.71\5.98  5.16\5.29  5.26\5.75  5.01\5.27 | 0.94\1.52  0.98\1.99  2.65\2.88  1.39\2.89  3.37\3.91  1.50\1.63  2.59\3.92  0.96\2.08 | 7.07\7.46  7.01\6.99  7.86\7.94  8.41\9.07  7.89\7.89  8.36\8.44  6.89\7.35  8.56\8.07 | 1.11\0.98  1.05\0.78  0.92\0.78  1.22\1.15  0.81\0.83  1.26\1.36  0.83\0.61  1.49\1.11 |
| EV_V6 | **EIN/2017**  **EIN/2018**  **ROG/2017**  **ROG/2018**  **GOL/2017**  **GOL/2018**  **TOM/2017**  **TOM/2018** | 5.03\5.54  4.73\4.73  5.55\5.91  6.14\6.42  6.24\6.24  5.12\4.77  5.58\5.86  6.30\5.43 | 0.97\1.51  1.07\2.58  1.02\2.52  2.21\3.36  3.90\3.81  1.21\1.17  2.96\3.90  2.44\1.07 | 8.05\8.39  6.95\6.08  8.07\7.76  8.81\9.68  8.45\7.94  8.23\7.51  7.66\7.91  9.60\9.08 | 1.24\1.06  0.78\0.56  0.95\0.77  1.29\1.20  0.85\0.85  1.29\1.26  0.92\0.68  1.36\1.26 |
| PH_V4 | **EIN/2017**  **EIN/2018**  **ROG/2017**  **ROG/2018**  **GOL/2017**  **GOL/2018**  **TOM/2017**  **TOM/2018** | 34.49\38.73  32.54\35.23  25.50\28.10  29.08\31.75  62.88\68.98  60.37\64.49  41.60\47.45  60.32\66.49 | 6.90\20.43  8.48\19.60  9.23\13.63  11.11\17.45  34.30\38.39  23.27\16.14  11.98\25.37  28.43\47.45 | 53.14\57.94  49.63\50.29  42.29\41.54  43.70\45.04  88.24\95.30  89.24\93.20  63.89\72.12  84.24\88.27 | 7.24\6.17  5.65\5.24  4.60\4.53  4.79\4.92  9.79\10.96  12.54\14.32  8.71\8.27  9.59\8.03 |
| PH_V6 | **EIN/2017**  **EIN/2018**  **ROG/2017**  **ROG/2018**  **GOL/2017**  **GOL/2018**  **TOM/2017**  **TOM/2018** | 62.40\69.36  78.81\85.14  61.46\68.91  82.64\90.89  94.21\98.30  101.90\104.82  83.86\92.35  120.46\120.57 | 21.41\36.53  21.90\52.02  32.17\30.35  41.48\57.90  37.28\54.75  53.69\50.37  48.46\57.81  68.48\58.96 | 95.54\98.80  105.05\115.61  89.74\94.77  118.69\123.27  127.54\130.51  137.67\146.02  119.07\124.98  173.66\169.71 | 11.89\9.62  10.53\10.07  9.34\9.52  11.00\11.17  15.05\15.29  15.42\18.24  14.41\12.79  19.56\18.63 |
| PH_final | **EIN/2017**  **EIN/2018**  **ROG/2017**  **ROG/2018**  **GOL/2017**  **GOL/2018**  **TOM/2017**  **TOM/2018** | 159.18\141.35  114.90\93.35  137.04\122.25  163.71\142.70  115.68\102.69  129.94\117.16  157.99\144.61  184.54\169.57 | 100.84\69.01  82.28\49.97  74.25\63.56  103.82\70.16  49.27\30.21  35.41\35.76  81.92\79.28  115.10\118.15 | 228.96\211.14  172.12\136.25  211.14\201.92  249.35\208.81  167.58\149.14  186.09\173.10  245.00\195.36  265.02\248.43 | 21.57\21.10  16.46\16.27  22.32\20.56  25.52\23.66  21.73\23.59  26.35\27.58  24.82\18.95  26.34\22.77 |
| FF | **EIN/2017**  **EIN/2018**  **ROG/2017**  **ROG/2018**  **TOM/2017**  **TOM/2018** | 82.55\81.78  78.80\79.59  73.06\71.91  79.16\79.05  76.88\74.16  70.31\68.76 | 70.36\68.86  63.37\68.12  62.45\59.10  66.74\67.74  63.93\62.13  62.22\60.14 | 102.02\101.50  94.35\93.96  91.22\88.03  100.14\92.87  93.28\92.17  83.64\90.06 | 5.23\5.17  5.44\5.40  4.82\4.47  4.72\4.41  5.58\4.64  4.11\3.73 |
| RL | **EIN/2017**  **EIN/2018**  **ROG/2017**  **ROG/2018** | 3.48\2.23  1.58\1.25  2.39\1.50  1.27\1.17 | 0.63\0.76  0.73\0.32  0.96\0.95  0.95\0.95 | 9.21\8.08  8.52\4.69  9.01\8.50  7.01\3.52 | 2.29\1.54  1.10\0.59  2.21\1.13  0.61\0.41 |
