# Supplementary material for: Genomic prediction using information across years with epistatic models and dimension reduction via haplotype blocks
Source: PLoS One. 2023 Mar 31;18(3):e0282288. doi: 10.1371/journal.pone.0282288 (PMC10065328; doi:10.1371/journal.pone.0282288)
Supplement: S2 Table — (DOCX) [file pone.0282288.s023.docx]

**S2** **Table.** The traits heritabilities in 2017, 2018 and both years jointly in KE (blue numbers) and PE (red numbers).

| Traits | 2017 | 2018 | Both 2017 and 2018 |
| --- | --- | --- | --- |
| EV_V3 | 0.91 / 0.85 | 0.79 / 0.67 | 0.92 / 0.86 |
| EV_V4 | 0.90 / 0.82 | 0.83 / 0.71 | 0.91 / 0.84 |
| EV_V6 | 0.88 / 0.84 | 0.75 / 0.68 | 0.90 / 0.85 |
| PH_V4 | 0.89 / 0.82 | 0.81 / 0.72 | 0.92 / 0.87 |
| PH_V6 | 0.90 / 0.88 | 0.85 / 0.81 | 0.93 / 0.91 |
| PH_final | 0.91 / 0.93 | 0.83 / 0.85 | 0.94 / 0.94 |
| FF | 0.90 / 0.92 | 0.86 / 0.83 | 0.94 / 0.93 |
| RL | 0.78 / 0.55 | 0.49 / 0.29 | 0.80 / 0.59 |
