# Supplementary material for: Genomic prediction using information across years with epistatic models and dimension reduction via haplotype blocks
Source: PLoS One. 2023 Mar 31;18(3):e0282288. doi: 10.1371/journal.pone.0282288 (PMC10065328; doi:10.1371/journal.pone.0282288)
Supplement: S3 Table — The stars represent the non-convergence of pre-estimated variance components based on the full set. (DOCX) [file pone.0282288.s024.docx]

**S3** **Table.** The percentage of bivariate models converged in 5-fold cross validation with 5 replicates based on pruned set of SNPs for both KE and PE (black percentages), only KE (blue percentages) and only PE (red percentages). The stars represent the non-convergence of pre-estimated variance components based on the full set.

| Traits | Predicted  Environments | GBLUP | ERRBLUP | sERRBLUP  Top 10% | sERRBLUP  Top 5% | sERRBLUP  Top 1% | sERRBLUP  Top 0.1% | sERRBLUP  Top 0.01% | sERRBLUP  Top 0.001% |
| --- | --- | --- | --- | --- | --- | --- | --- | --- | --- |
| EV_V3 | **ROG** | 92% | 88% | 96% | 88% | 52%* | 68%* | 60%* | 16%* |
|  | **GOL** | 100% | 100% | 0%* | 0%* | 100% | 72% | 84% / 44% | 1 / 60% |
|  | **TOM** | 100% | 100% | 92% | 96% | 100% | 96% / 96% | 92% / 64% | 40%* / 28%* |
|  |  |  |  |  |  |  |  |  |  |
|  | **EIN** | 100% | 100% | 100% | 100% | 100% | 80% / 100%* | 76% | 100% |
| EV_V4 | **ROG** | 96% | 96% | 100% | 100% | 96% | 100% | 96% / 96% | 76% / 96% |
|  | **GOL** | 100% | 100% | 100% | 100% | 100% | 100%* | 100% | 68% |
|  | **TOM** | 40%* / 96% | 52% / 96% | 88% | 100% | 96% | 100%* | 100% | 96% |
|  |  |  |  |  |  |  |  |  |  |
|  | **EIN** | 100% | 100% | 100% | 100% | 100% | 96% | 96% | 100%*/ 96% |
| EV_V6 | **ROG** | 100% | 100% | 100% | 100% | 100% | 100% | 56%* / 92% | 0%* |
|  | **GOL** | 100% | 100% | 0%* / 0%* | 0% / 0%* | 0%* | 16%* / 0%* | 0%* | 100% |
|  | **TOM** | 100% | 100% | 100% | 100% | 100% | 100% | 100% | 100% |
|  |  |  |  |  |  |  |  |  |  |
|  | **EIN** | 100% | 100% | 100% | 96% | 100% | 100% | 96% | 100% |
| PH_V4 | **ROG** | 100% | 100% | 100% | 100% | 100% | 84% | 84% | 92% |
|  | **GOL** | 100% | 100% | 0%* | 0%* | 0%* | 12%* | 100% | 100% |
|  | **TOM** | 96% / 92% | 96% / 96% | 100% | 100% | 100% | 92% | 92% | 96% |
|  |  |  |  |  |  |  |  |  |  |
|  | **EIN** | 100% | 100% | 100% | 100% | 100% | 100% | 100% | 100% |
| PH_V6 | **ROG** | 100% | 100% | 100% | 100% | 100% | 84% | 92% | 84%* |
|  | **GOL** | 96% | 96% | 96% | 96% | 100% | 100% | 96% | 84%* |
|  | **TOM** | 100% | 100% | 100% | 100% | 100% | 100% | 96% | 100% |
|  |  |  |  |  |  |  |  |  |  |
|  | **EIN** | 100% | 100% | 100% | 100% | 96% | 84% | 76%* | 72%* |
| PH_final | **ROG** | 100% | 100% | 0%* / 88% | 0%* / 0%* | 0%* / 0%* | 0% */ 0%* | 92% / 0%* | 48%* |
|  | **GOL** | 100% | 100% | 100% | 100% | 96% | 100% | 100% | 68% |
|  | **TOM** | 100% | 100% | 4%* / 96% | 92% | 20% */ 96% | 4%* / 68%* | 96%* / 52%* | 76% |
|  |  |  |  |  |  |  |  |  |  |
|  | **EIN** | 100% | 100% | 96% | 100% | 64% | 0%* | 64% | 92% |
| FF | **ROG** | 100% | 100% | 96% / 0%* | 0%* | 96% / 0%* | 4%* / 0%* | 56%* / 0%* | 24%* |
|  | **TOM** | 88% / 80% | 64%* / 84% | 96% / 80% | 96% / 80% | 92% / 76% | 60% / 52%* | 36%* / 32%* | 76% / 72% |
|  |  |  |  |  |  |  |  |  |  |
| RL | **EIN** | 100% | 100% | 100% | 100% | 100% | 100% | 100% | 100% |
|  | **ROG** | 100% | 100% | 100% | 100% | 96% | 92% | 72% | 84% |
