# Supplementary material for: Genomic prediction using information across years with epistatic models and dimension reduction via haplotype blocks
Source: PLoS One. 2023 Mar 31;18(3):e0282288. doi: 10.1371/journal.pone.0282288 (PMC10065328; doi:10.1371/journal.pone.0282288)
Supplement: S4 Table — (DOCX) [file pone.0282288.s025.docx]

**S4 Table.** The number of epistasis interactions maintained in the model based on haplotype blocks and pruned set of SNPs for each proportions of interactions in KE and PE.

| Proportions of interactions | Landrace | Pruned set of SNPs | Haplotype Blocks |
| --- | --- | --- | --- |
| 100% | **KE** | 323'533'203 | 4'416'392 |
|  | **PE** | 456'397'578 | 5'546'115 |
| Top 10% | **KE** | 32'353'320 | 441'639 |
|  | **PE** | 45'639'758 | 554'612 |
| Top 5% | **KE** | 16'176'660 | 220'820 |
|  | **PE** | 22'819'879 | 277'306 |
| Top 1% | **KE** | 3'235'332 | 44'164 |
|  | **PE** | 4'563'976 | 55'461 |
| Top 0.1% | **KE** | 323'533 | 4'416 |
|  | **PE** | 456'398 | 5'546 |
| Top 0.01% | **KE** | 32'353 | 443 |
|  | **PE** | 45'640 | 555 |
| Top 0.001% | **KE** | 3'235 | 44 |
|  | **PE** | 4'564 | 55 |
