# Supplementary material for: Genomic prediction using information across years with epistatic models and dimension reduction via haplotype blocks
Source: PLoS One. 2023 Mar 31;18(3):e0282288. doi: 10.1371/journal.pone.0282288 (PMC10065328; doi:10.1371/journal.pone.0282288)
Supplement: S6 Table — (DOCX) [file pone.0282288.s027.docx]

**S6** **Table.** GBLUP predictive ability based on pruned set of SNPs for prediction in 2018 with training the model either on 2018 data or the average phenotypic values of 2017 and 2018 in each environment for series of phenotypic traits in PE.

| Trait | Training set | EIN | ROG | GOL | TOM |
| --- | --- | --- | --- | --- | --- |
| EV_V3 | 2018  2017 and 2018 average | NA  NA | 0.209  0.210 | 0.337  0.332 | 0.326  0.323 |
| EV _V4 | 2018  2017 and 2018 average | 0.185  0.205 | 0.261  0.269 | 0.625  0.646 | 0.363  0.371 |
| EV _V6 | 2018  2017 and 2018 average | 0.323  0.288 | 0.362  0.382 | 0.627  0.645 | 0.583  0.572 |
| PH_V4 | 2018  2017 and 2018 average | 0.464  0.480 | 0.510  0.396 | 0.641  0.661 | 0.437  0.439 |
| PH_V6 | 2018  2017 and 2018 average | 0.547  0.541 | 0.529  0.440 | 0.690  0.706 | 0.515  0.512 |
| PH_final | 2018  2017 and 2018 average | 0.620  0.547 | 0.537  0.370 | 0.682  0.699 | 0.580  0.572 |
| FF | 2018  2017 and 2018 average | 0.466  0.495 | 0.456  0.079 | 0.306  - | 0.401  0.363 |
| RL | 2018  2017 and 2018 average | 0.268  0.235 | 0.419  0.114 | -  - | -  - |
