# Supplementary material for: Genomic prediction using information across years with epistatic models and dimension reduction via haplotype blocks
Source: PLoS One. 2023 Mar 31;18(3):e0282288. doi: 10.1371/journal.pone.0282288 (PMC10065328; doi:10.1371/journal.pone.0282288)
Supplement: S15 Table — The blue and red bold numbers with stars indicate which proportion of interactions in bivariate sERRBLUP maximized the predictive ability based on pruned set of SNPs in each environment for KE and PE, respectively. (DOCX) [file pone.0282288.s036.docx]

**S15** **Table.** Genomic correlation between 2017 and 2018 in each environment for trait EV_V4 for KE (blue numbers) and PE (red numbers). The blue and red bold numbers with stars indicate which proportion of interactions in bivariate sERRBLUP maximized the predictive ability based on pruned set of SNPs in each environment for KE and PE, respectively.

| Bivariate Models | EIN | ROG | GOL | TOM |
| --- | --- | --- | --- | --- |
| GBLUP | 0.843 / 0.525 | 0.897 / 0.592 | 0.940 / 0.998 | 0.960 / 0.835 |
| sERRBLUP top 10% | **0.810*** / 0.713 | **0.863*** / 0.618 | 0.872 / 0.815 | 0.972 / **0.744*** |
| sERRBLUP top 5% | 0.807 / 0.732 | 0.861 / 0.632 | 0.830 / **0.782*** | 0.997 / 0.751 |
| sERRBLUP top 1% | 0.865 / 0.769 | 0.889 / **0.705*** | **0.791*** / 0.824 | 0.941 / 0.733 |
| sERRBLUP top 0.1% | 0.966 / 0.888 | 0.936 / 0.766 | 0.874 / 0.946 | 0.934 / 0.784 |
| sERRBLUP top 0.01% | 0.979 / 0.908 | 0.947 / 0.780 | 0.883 / 0.999 | 0.979 / 0.806 |
| sERRBLUP top 0.001% | 0.974 / **0.898*** | 0.966 / 0.758 | 0.861 / 0.932 | **0.999*** / 0.834 |
