# Supplementary material for: Genomic prediction using information across years with epistatic models and dimension reduction via haplotype blocks
Source: PLoS One. 2023 Mar 31;18(3):e0282288. doi: 10.1371/journal.pone.0282288 (PMC10065328; doi:10.1371/journal.pone.0282288)
Supplement: S21 Table — (DOCX) [file pone.0282288.s042.docx]

**S21 Table.** Phenotypic correlation between 2017 and 2018 in each environment for KE and PE.

| Trait | Landrace | EIN | ROG | GOL | TOM |
| --- | --- | --- | --- | --- | --- |
| EV_V3 | **KE**  **PE** | -  - | 0.481 0.373 | 0.679  0.510 | 0.623  0.523 |
| EV_V4 | **KE**  **PE** | 0.553  0.341 | 0.587  0.396 | 0.709  0.725 | 0.570  0.495 |
| EV_V6 | **KE**  **PE** | 0.551 0.350 | 0.583  0.454 | 0.729  0.749 | 0.383  0.414 |
| PH_V4 | **KE**  **PE** | 0.736 0.641 | 0.646  0.504 | 0.779  0.793 | 0.566  0.599 |
| PH_V6 | **KE**  **PE** | 0.746 0.650 | 0.681 0.545 | 0.781  0.845 | 0.662  0.508 |
| PH_final | **KE**  **PE** | 0.688 0.668 | 0.768 0.739 | 0.721  0.790 | 0.778  0.771 |
| FF | **KE**  **PE** | 0.656 0.650 | 0.780 0.743 | -  - | 0.745  0.639 |
| RL | **KE**  **PE** | -  - | 0.436  0.226 | -  - | 0.377  0.329 |
